# Effectiveness of Very Brief Advice on Tobacco Cessation: A Systematic Review and Meta-Analysis

**DOI:** 10.1007/s11606-024-08786-8

**Published:** 2024-05-02

**Authors:** Christopher Chi Wai Cheng, Wan Jia Aaron He, Hebe Gouda, Min Jin Zhang, Tzu Tsun Luk, Man Ping Wang, Tai Hing Lam, Sophia Siu Chee Chan, Yee Tak Derek Cheung

**Affiliations:** 1 School of Nursing, the University of Hong Kong, Hong Kong, China; 2 School of Public Health, University of Queensland, Brisbane, QLD Australia; 3 World Health Organization, Geneva, Switzerland; 4 School of Public Health, the University of Hong Kong, Hong Kong, China

**Keywords:** smoking cessation, very brief advice, tobacco

## Abstract

**Background:**

Very brief advice (VBA; ≤ 3 min) on quitting is practical and scalable during brief medical interactions with patients who smoke. This study aims to synthesize the effectiveness of VBA for smoking cessation and summarize the implementation strategies.

**Methods:**

We searched randomized controlled trials aiming at tobacco abstinence and comparing VBA versus no smoking advice or no contact from Medline, Embase, CINAHL, Cochrane Library, PsycInfo databases, six Chinese databases, two trial registries ClinicalTrials.gov and WHO-ICTRP from inception to September 30, 2023. Grading of Recommendations, Assessment, Development, and Evaluations framework was used to assess the certainty of the evidence of the meta-analytic findings. The outcomes were self-reported long-term tobacco abstinence at least 6 months after treatment initiation, earlier than 6 months after treatment initiation, and quit attempts. Effect sizes were computed as risk ratio (RR) with 95% CI using frequentist random-effect models.

**Data Synthesis:**

Thirteen randomized controlled trials from 15 articles (*n* = 26,437) were included. There was moderate-certainty evidence that VBA significantly increased self-reported tobacco abstinence at ≥ 6 months in the adjusted model (adjusted risk ratio ARR 1.17, 95% CI: 1.07–1.27) compared with controls. The sensitivity analysis showed similar results when abstinence was verified by biochemical validation (*n* = 6 studies, RR 1.53, 95% CI 0.98–2.40). There was high-certainty evidence that VBA significantly increased abstinence at < 6 months (ARR 1.22, 95% CI: 1.01–1.47). Evidence of effect on quit attempts (ARR 1.03, 95% CI 0.97–1.08) was of very low certainty.

**Discussion:**

VBA delivered in a clinical setting is effective in increasing self-reported tobacco abstinence, which provides support for wider adoption in clinical practice.

**Supplementary Information:**

The online version contains supplementary material available at 10.1007/s11606-024-08786-8.

## BACKGROUND

Tobacco cessation prevents premature death, many types of cancers and cardiovascular and pulmonary diseases.^1^ However, many smokers have not received advice to quit, and only few quit attempters have used tobacco cessation treatment.^2–4^ In low- and middle-income countries, only 40% of smokers received advice to quit smoking from healthcare providers in the past year.^2^ On the other hand, about 176.8 million adults in 31 countries made a quit attempt in the past 12 months.^3^ Only about 10% of quit attempters had used tobacco cessation aids, including counseling and medications, in 15 countries,^4^ probably because of low accessibility or awareness of these services in smokers.^5,6^

Brief intervention (BI) aims to deliver an evidence-based smoking cessation (SC) intervention within a minimal time period by identifying smokers, advising, and assisting them to quit.^7,8^ BI was effective in directing patients to SC treatments and increasing tobacco abstinence.^9–13^ The 5As (Ask, Advise, Assess, Assist, Arrange follow-up) and 5Rs (Relevance, Risk, Rewards, Roadblocks, Repetition) model are the most known BI models recommended by the World Health Organization (WHO),^14^ the US Centers for Disease Control and Prevention,^15^ and the 2020 Surgeon General Report.^1^ However, many healthcare providers cannot adhere to or implement the full BI.^16^ More simplified SC intervention models, namely very brief advice (VBA), such as AAR (Ask, Advise, Refer),^17^ AWARD (Ask, Warn, Advice, Refer, Do-it-again),^8^ and the ABC (Ask about smoking, give Brief advice to quit, and offer Cessation assistance)^18^ were developed for healthcare practitioners to implement easily in routine medical consultation, with very short duration. While BI acts primarily by motivating quit attempts and delivering quitting aids, VBA acts by giving opportunistic advice to all smokers, irrespective of motivation to quit. For instance, New Zealand’s clinical guideline adopts a proactive “opt-out” ABC approach to provide very brief opportunistic advice to all smokers without a preliminary assessment of willingness to quit.^18^

Two contextual factors inherent to clinical practice facilitate the implementation of VBA over BI by healthcare providers. First, clinical settings that have time constraints and short consultations may allow only very brief (1–2 min) discussions on smoking cessation. Assessment of motivation as required by “opt-in approaches” is time-consuming and often not feasible.^19^ Second, not all healthcare professionals are trained or specialized in tobacco cessation and counseling skills to deliver more intensive treatment. Therefore, VBA is probably the most convenient model to advise and refer patients who smoke to use SC services.^20^

Some randomized controlled trials have tested the effectiveness of VBA) as short as 30 s and supported its effectiveness.^21,22^ VBA may help reduce the barriers of the increased time demand on healthcare workers in providing SC advice and tackle the impracticality of longer interventions in busy clinical settings. General practitioners trained with the ABC model delivered more SC advice after training than those trained with conventional 5As (between group: 35.7% vs 30.3%, adjusted odds ratio 1.71, 95% CI 0.94–3.12).^23^ Both healthcare providers and patients preferred VBA for SC due to its feasibility, simplicity, and ease.^21,24^ BI was commonly defined as taking 10 to 30 min in systematic reviews.^10–12^ Some systematic reviews even included trials testing “BI” which exceeded 30 min,^9,13^ which far exceeded the usual time (about 10 min) for medical consultation. However, no previous systematic reviews that specifically synthesized the RCTs result in the effectiveness of opportunistic SC advice that was 3 min or less. Thus, we aimed to build upon the previous review by Aveyard et al. (2012) to (i) synthesize the effectiveness of VBA on SC to better assess the specific effect of VBA in 3 min or less without other intensive intervention components and (ii) summarize the implementation strategies and settings of VBA to recognize the contextual factors for implementation.

## METHODS

### Data Sources and Searches

The study was registered with The International Prospective Register of Systematic Reviews (PROSPERO) (Ref No: CRD42022341466). Based on the Preferred Reporting Items for Systematic Review and Meta-Analyses (PRISMA) checklist,^25^ we elaborated the question of the review in terms of Population, Intervention, Comparison, Outcomes, Study Designs (PICOS), to search systematically: “What is the effectiveness of the VBA on the long-term tobacco abstinence in current (daily or occasional) tobacco users including cigarettes, smokeless tobacco, heated tobacco products, and cigars, compared with no SC advice or no contact from randomized controlled trials?” Medline, Embase, CINAHL, Cochrane Library, PsycInfo databases, two trial registries ClinicalTrials.gov and WHO-ICTRP, and additional six Chinese databases were systematically searched from inception to September 30, 2023. The full search strategy is shown in the eMethods of Supplement 1.

### Study Selection

We included studies with the following criteria: (1) individual or cluster randomized controlled trials, (2) long-term tobacco abstinence outcomes were reported, (3) behavioral intervention duration was 3 min or less as stated in manuscripts or judged by assessors, and (4) the control group received no SC advice or no contact. In studies with intervention arm(s) involving pharmacotherapy, we included them if their data from non-pharmacotherapy trial arms can be extracted. We also excluded studies (1) if their number of follow-up interventions was more than twice per month, as such follow-up interventions could have a stronger effect than VBA in the first contact, and (2) recruiting ex-smokers, people currently attempting to quit or non-tobacco users including such as electronic nicotine delivery systems (ENDS). We had no restriction on languages used, but an abstract written in English or Chinese was required. While eligibility of a non-English or non-Chinese full-text was being assessed, a translator would assist in evaluating the study. Eventually, we did not identify any such studies. We had no restrictions on study setting, age limit of the participants, and types of intervention.

### Data Extraction and Outcomes

The relevant data were extracted independently by two co-authors (CCCW and WJAH). The primary outcome was self-reported tobacco abstinence at ≥ 6 months after treatment initiation. Assessing posttreatment initiation of abstinence was preferred compared to the target quit day as smokers might or might not have stopped smoking on the target quit day.^26^ We used the commonly adopted minimal time of follow-up for the assessment of tobacco cessation, which is 6 months after intervention. Continuous abstinence was used if both continuous and point prevalence results were available. Self-report smoking status was chosen over biochemical validation of abstinence because some RCTs did not validate abstinence with biochemical methods. Also, the process of inviting smokers to come back for biochemical validation could have motivated some to quit smoking for a few days before validation and often includes monetary incentives, which could increase abstinence. Nevertheless, we performed a sensitivity analysis to examine whether the pooled estimates of self-reported abstinence produced similar results as those verified by biochemical validation.

Secondary outcomes included tobacco abstinence at < 6 months after treatment initiation and quit attempts. “Quit attempts” was defined as at least one attempt to stop using tobacco products lasting for 24 h or more.^27^ Risk ratios were extracted when available or calculated based on the reported descriptive statistics.

### Quality Assessment

We used the GRADE approach to evaluate the overall certainty of evidence for key outcomes based on study risk of bias, inconsistency of results, indirectness of evidence, imprecision, and publication bias.^28^ Sensitivity analyses were done by moderation analysis based on the risk of bias obtained by the Cochrane Risk of Bias Assessment Tool, particularly the items related to the exchangeability of treatment and control group, random sequence generation, and allocation concealment. Inconsistency was assessed by Cochran’s Q test in the overall estimate and test of heterogeneity in subgroup analysis. Indirectness was assessed by whether the outcome of interest was measured differently from recommendations or guidelines. Imprecision was assessed by the effect estimate in relation to the null effect.^28^ A funnel plot was used to assess publication bias with Egger’s regression and significance level of *P* < 0.1.^29^ When publication bias was detected, we further adjusted for the missing studies by non-prespecified Duval and Tweedie’s “trim and fill” analysis and estimated the numbers and outcomes of missing studies.^30^

### Data Synthesis

Clinical heterogeneities were assessed by tabulating the study characteristics, including delivery settings (e.g., inpatient, outpatient, community), interventionists (i.e., profession to deliver the intervention), use of intervention models (i.e., any specific advice model adopted in the study), duration of the advice, use of self-help materials, and any relevant features where available.

Two co-authors (CCCW and WJAH) evaluated the risk of bias independently for the included studies using the Cochrane collaboration risk of bias tool.^31^ Any discrepancies were resolved by discussion with a third co-author (DCYT). A stacked bar chart was used to show the categories of (1) high risk, (2) unclear risk, (3) low risk in each domain included in the risk of bias tool.

To check the comparability of the studies before pooling the outcome data in the meta-analysis, we conducted tests for moderation to assess the potential influence of differences in randomization generation, conflict of interest, and follow-up time points across studies. When there is no evidence of moderation from these factors, the data from the included studies were pooled to generate a robust estimate of the overall treatment effect.

### Quantitative Analysis

Data analyses were done by R, with “metafor” package.^32^ We used random-effect frequentist meta-analytic models to analyse the tobacco abstinence outcomes, with a significance level of *P* < 0.05. We used the inverse variance weighting method to pool the result of the combined study effect for risk ratios and 95% confidence interval (CI) when available. The number needed to treat (NNT) was estimated by the reciprocal of the risk difference. The results were visualized by a forest plot, showing weights and publication year, individual and pooled effect estimates and 95% CI.

### Subgroup Analysis

We did subgroup analyses on certain study characteristics of age, high- vs low-income countries and types of interventionists when there were more than two available studies. We used Cochran’s Q test to examine the heterogeneity quantitatively with a significance level of *P* < 0.1.^33^ The I-squared statistics by Higgins and Thompson were used to quantify heterogeneity.^34^

## RESULTS

### Study and Participant Characteristics

**Figure 1:**
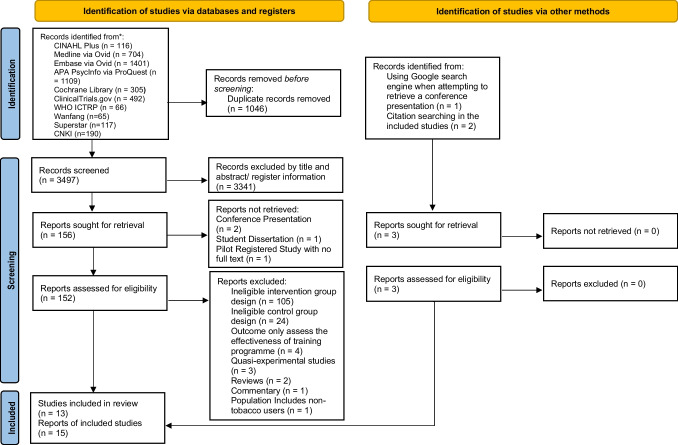
PRISMA Flowchart for study selection. *No studies can be found in Airiti Library, Taiwan Periodical Literature and Government Research Bulletin.

A total of 13 RCTs (no cluster RCTs) from 15 articles (*n* = 26,437) published from 1979 to 2021 were included in the synthesis^35–49^ (Fig. 1). The extracted general information and abstinence results in each study are presented in Tables 1 and 2, respectively. More details are also shown in eResults 1.

**Table 1:** Study and Intervention Design

First author (year)	Sample size	City and associated Country	Population	Intervention framework (Message, aids, follow-up)	Intervention duration	Delivery personnel and their capacity building	Control content	Setting(s)
Russell (1979)^45^	2138	London, United Kingdom	^3^16 y/o, British, current cigarette smokers, outpatient (GP) clinic patients that see a doctor	Message: Given in doctors’ own style Aids: Some were given a smoking cessation leaflet and being warned of a follow-up Follow-up: No follow-up Intervention	1 to 2 min (No compliance check)	Physicians Preparation: not stated	Usual care^a^	Outpatient clinic
Russell (1983)^46^	1377	London and Kent, United Kingdom	^3^16 y/o, British, current cigarette smokers, outpatient (GP) clinic patients that see a doctor	Message: Given in doctors’ own style Aids: All received a smoking cessation leaflet and being warned of a follow-up Follow-up: No follow-up Intervention	1 to 2 min (No compliance check)	Physicians Preparation: not stated	Usual care^a^	Outpatient clinic
Jamrozik (1984)^40^	1061	London, United Kingdom	^3^16 y/o, British, currently smoking cigarette (exclude pipe/cigar)	Message: Given in doctors’ own style Aids: All received a smoking cessation leaflet and being warned of a follow-up Follow-up: No follow-up interventions	1 to 2 min (No compliance check)	Physicians Preparation: not stated	Usual care^a^	Outpatient clinics
Folsom (1987)^39^	258	Minneapolis-St. Paul, United States	Age range not specified; cigarette smokers (checked by nurse)	Message: Standardized messages of which indicate smoking is a major cause of death and that the participant should quit (not stated further about the content) Aid: Nil Follow-up: Nil	1 to 2 min (No compliance check)	Physicians Preparation: not stated	Usual care^a^	Outpatient clinic of a large health maintenance organization
Slama (1990)^48^	311	Newcastle, Australia	18–64 y/o, self-reported smokers (not specified types of tobacco)	Message: framework and content not provided Aids: All received 3 Smoking cessation brochures Follow-up: No follow-up Intervention	1.4 min (No compliance check)	Physicians Preparation: 1-h training workshop to help physicians get familiar with the intervention procedures	Usual Care^a^	Unspecified clinical setting (Only specified the intervention delivered by general practices to patients)
Severson (1998)^47^	2637	Oregon, United States	^3^15 y/o, current cigarette smokers	Message: direct advice related to oral health with protocol Aids: All received health education leaflets; a kit containing gums, candy, rubber bands that helped with smoking cessation Follow-up: No follow-up Intervention	Not stated (Anticipated less than 3 min) (No compliance check)	Dentist and dental hygienists Preparation: training workshop to familiar with the intervention procedures	Usual Care^a^	Dental clinic
Betson (2000)^35^ Lam (2000)^36^	865	Hong Kong, China	15–65 y/o, Chinese (HK), current cigarette users, outpatient clinic patients (old and new cases)	Message: Shortened standardized advice adaptation from 4A (Ask, Advice, Assist and Arrange) Aids: Some were given a self-help smoking cessation booklet Follow-up: No follow-up interventions	1 min (No compliance check)	Physicians Preparation: briefing with verbal and written instruction	Some received a smoking cessation booklet and some had no intervention	Government general outpatient clinics
Loke (2005)^44^	758	Guangzhou, China	^3^21 y/o; Married male cigarette smokers with a non-smoking wife living in the same household	Message: Standardized brief advice considered to be realistic in the prenatal clinic informed by Fishbein & Ajzen theory of reasoned action (creditability of knowledge, severity of the consequences of their behavior, motivation, support of significant others, and empowerment) Aids: All received a health education booklet Follow-up: at least 2 standardized face-to-face health reminders	Initial advice: 2–3 min Booster Reminders: 90 s (No compliance check)	Physicians Preparation: workshop training and briefing to familiarize the intervention procedures	Usual care^a^	Prenatal outpatient clinic
Lin (2013)^42,43^	126	Guangzhou, China	Age range not specified; current cigarette smokers	Message: Standardized message with script; WAR model (Warn, Advice, Refer) Aids: Nil Follow-up: Nil	20–30 s (No compliance check)	Physicians Preparation: workshop training less than 1 h and briefing to familiarize the intervention procedures	Usual care^a^	Outpatient clinic of internal medicine in the hospital
Wu (2017)^49^	369	Beijing, China	^3^18 y/o, Chinese, currently smoking ^3^10 cigarette per day in past month, no intention to quit smoking	Message: standardized script with the instruction to reduce tobacco consumption Aids: Nil Follow-up: 1-min intervention booster calls at each follow-up (1 week and 1, 3, 6 and 12 months)	1 min (With compliance checked by the research team)	Physicians Preparation: training workshop to familiarize with the intervention procedures	Very brief (1 min) exercise and diet advice 1 min placebo booster calls at each follow-up that provide exercise and diet advice	Acupuncture and endocrinology outpatient clinic
Cheung (2018)^37^	1295	Vancouver, Canada	^3^19 y/o; current tobacco smokers (mainly cigarettes, with few using cigars and other types)	Message: Brief counseling based on Ask, Advice, and Refer with standardized script Aids: All received a leaflet on available smoking cessation services and an offer to refer to a smoking cessation service Follow-up: No follow-up Interventions	Less than 30 s (No compliance check)	Physicians Preparation: not stated	Usual care^a^	Hospital emergency departments
Li (2020)^41^	1571	Hong Kong, China	^3^18 y/o, Chinese (HK), current cigarette smokers, emergency department patients, not receiving cessation treatment, triage as semi-urgent/non-urgent	Message: Standardized message with script; AWARD model (Ask, Warn, Advice, Refer, Do-it-again) Aids: Informed by self-determination theory, smokers chose their quit schedules (immediate or progressive) and received leaflets that have relevant advice Follow-up: 1 to 2 min intervention booster call at each follow-up (1, 3, 6, and 12 months)	Initial advice: 1 min Booster: 1 to 2 min (Compliance check by audiotaping)	Nurses Preparation: workshop training with materials (no clear details)	Smoking cessation leaflet; 1 min placebo booster calls at each follow-up that promote physical activity, fruit, and vegetable intake	Hospital emergency department
Cheung (2021)^38^	13,671	Guangdong, China	^3^18 y/o, Chinese, currently smoking ^3^1 cigarette per day, not receiving cessation treatment	Message: Standardized scripted messages based on Warn, Advise, Refer (WAR) Aids: Leaflet and card that contained motivational messages and provide contact of smoking cessation clinic Follow-up: Some received a booster intervention at 1-month follow-up	30 s for both initial and booster (With compliance check with a physicians’ survey)	Physicians Preparation: 1-h training workshop on smoking cessation	Very brief (30 s) advice about fruit and vegetable consumption with relevant leaflet and card	Hospital outpatient clinics

^a^Usual care was defined as no smoking cessation advice or no contact

**Table 2:** Measurements and Findings

First author (year)	abstinence at < 6 months		abstinence at ≥ 6 months		biochemical validation for abstinence at ≥ 6 months		Quit attempts	
	Measurement	Findings	Measurement	Findings	Measurement	Findings	Measurement	Findings
Russell (1979)^45^	1-month abstinence (Duration of abstinence was not specified)	Intervention: 5.4% (56/1031) Control: 2.4% (27/1107) RR: 2.23 (1.42, 3.50)	12-month SA at 12-month follow-up	Intervention: 3.3% (34/1031) Control: 0.7% (8/1107) RR: 4.56 (2.12, 9.81)	Salivary nicotine concentration (The threshold was not specified) at 12-month follow-up	Biochemical validation was not formally done between intervention and control group	At least once, attempted to quit at a 12-month follow-up	Intervention: 13.2% (62/471) Control: 9.7% (90/930) RR: 1.36 (1.004, 1.84)
Russell (1983)^46^	Self-reported abstinence at 4-month follow-up (Duration of abstinence was not specified)	Intervention: 12.8% (95/740) Control: 9.4% (60/637) RR: 1.36 (1.004, 1.85)	12-month SA at 12-month follow-up	Intervention: 5.8% (43/740) Control: 5.5% (35/637) RR: 1.06 (0.69, 1.63)	Carbon monoxide in expired air 7 ppm less than that in ambient air at 12-month follow-up	Intervention: 3.8% (28/740) Control: 3.6% (23/637) RR: 1.05 (0.61, 1.80)	At least once, attempted to quit at a 4-month follow-up	Intervention: 46.1% (311/675) Control: 36.6% (214/584) RR: 1.34 (1.17, 1.53)
Jamrozik (1984)^40^	N/A	N/A	12-month SA at 12-month follow-up	Intervention: 15.0% (77/512) Control: 10.6% (58/549) RR: 1.42 (1.03, 1.96)	Urinary cotinine lower than or equal to 100 ng/ml at 12-month follow-up	Intervention: 2.1% (11/512) Control: 0.73% (4/549) RR: 2.95 (0.94, 1.96)	Attempts to stop smoking (not specified how to measure quit attempt)	Cannot be retrieved because of incomplete outcome data
Folsom (1987)^39^	3-month abstinence (Duration of abstinence was not specified)	Intervention: 8.0% (11/137) Control: 5.8% (7/121) RR: 1.39 (0.56, 3.47)	N/A	N/A	N/A	N/A	At least one quit attempt for those who continue to smoke only at 3-month follow-up	Cannot be retrieved because of incomplete outcome data
Slama (1990)^48^	Abstinence at 1-month follow-up (Duration of abstinence was not specified)	Intervention: 14.4% (15/104) Control: 9.4% (10/106) RR: 1.53 (0.72, 3.25)	11-month SA at 12-month follow-up	Intervention: 2/104 (1.9%) Control:1/106 (0.94%) RR: 2.04 (0.19, 22.14)	Salivary cotinine less than 50 nmol/l at 12-month follow-up	Intervention: 1/104 (0.96%) Control:1/106 (0.94%) RR: 1.02 (0.064, 16.08)	N/A	N/A
Severson (1998)^47^	7-day PPA at 3-month follow-up	Intervention: 5.1% (66/1305) Control: 4.7% (63/1350) RR: 1.08 (0.77, 1.52)	9-month SA at 12-month follow-up	Intervention: 2.6% (34/1305) Control: 2.4% (32/1350) RR: 1.04 (0.83, 1.33)	N/A	N/A	At least one quit attempt at 12-month follow-up	Cannot be retrieved because of Incomplete outcome data
Betson (2000)^35^ Lam (2000)^36^	30-day PPA at 3-month follow-up	Intervention: 11.1% (49/443) Control: 8.3% (35/422) RR: 1.33 (0.88, 2.02)	9-month SA at 12-month follow-up	Intervention: 3.2% (14/443) Control: 3.1% (13/422) RR: 1.03 (0.49, 2.16)	N/A	N/A	N/A	N/A
Loke (2005)^44^	30-day PPA at 3-to-5-month follow-up	Intervention: 6.1% (23/380) Control: 4.2% (16/378) RR: 1.43 (0.77, 2.66)	N/A	N/A	N/A	N/A	Attempts to give up smoking in the last 7 days at 3-to-5-month follow-up	Intervention: 30% (114/380) Control: 22.2% (84/378) RR: 1.35 (1.06, 1.72)
Lin (2013)^42,43^	3-month SA at the 1 and 3-month follow-up	Intervention: 18.9% (14/74) Control: 3.8% (2/52) RR: 4.92 (1.17, 20.73)	11-month SA at 12-month follow-up	Intervention: 17.6% (11/74) Control: 3.8% (2/52) RR: 3.86 (0.89, 16.71)	N/A	N/A	N/A	N/A
Wu (2017)^49^	30-day PPA at 3-month follow-up	Intervention: 14.4% (26/181) Control: 6.9% (13/188) RR: 2.08 (1.10, 3.92)	6-month SA at 12-month follow-up	Intervention: 10.5% (19/181) Control: 5.3% (10/188) RR: 1.97 (0.94, 4.13)	Carbon monoxide in expired air less than 6 ppm at 12-month follow-up	Intervention: 10.5% (11/181) Control: 5.3% (4/188) RR: 2.86 (0.93, 8.81)	N/A	N/A
Cheung (2018)^37^	30-day PPA at 3-month follow-up	Intervention: 11.5% (76/660) Control: 11.5% (73/635) RR: 1.002 (0.74, 1.36)	30-day PPA at 12-month follow-up	Intervention: 14.4% (95/660) Control: 12.8% (81/635) RR: 1.13 (0.86, 1.49)	N/A	N/A	At least one 7-day quit attempt at 12-month follow-up	Intervention: 225/660 (34.1%) Control: 214/635 (33.7%) RR: 1.01 (0.87, 1.18)
Li (2020)^41^	N/A	N/A	7-day PPA at the 12-month follow-up	Intervention: 13.0% (102/787) Control: 8.5% (67/784) aRR: 1.46 (1.06, 2.19)	Carbon monoxide in expired air lower than 9 ppm and salivary cotinine lower than 115 ng/ml at 12-month follow-up	Intervention: 13.0% (55/787) Control: 8.5% (29/784) aRR: 2.23 (1.25, 3.97)	N/A	N/A
Cheung (2021)^38^	3-month 30-day PPA at 3-month follow-up	Intervention: 4.6% (321/7015) Control: 3.7% (247/6656) RR: 1.23 (1.05, 1.45)	30-day PPA at the 12-month follow-up	Intervention: 8.0% (559/7015) Control: 6.9% (458/6656) RR: 1.16 (1.03, 1.30)	Carbon monoxide in expired air less than 4 ppm and salivary cotinine lower than 10 ng/ml at 12-month follow-up	Intervention: 0.83% (58/7015) Control: 0.83% (55/6656) RR: 1.0006 (0.69, 1.44)	At least one 24-h quit attempt RR at 12-month follow-up	Intervention: 23.2% (1629/7015) Control: 22.6% (1502/6656) RR: 1.03 (0.97, 1.09)

*SA* sustained (continuous/prolonged) abstinence, *PPA* point prevalence abstinence, (a)RR = (adjusted) risk ratio/relative risk, 95% confidence interval given in bracket behind RR;

*ppm* parts per million, (a)RR = (adjusted) risk ratio/relative risk, 95% confidence interval given in bracket behind RR

### Intervention Strategies

Twelve studies recruited subjects in outpatient clinics (*n* = 10)^35,36, 38–40, 42–47, 49^ and emergency departments (*n* = 2),^37,41^ and one study did not specify the setting.^48^ Interventions were mainly delivered by physicians (*n* = 11)^35–40, 42–46, 48, 49^ and the others by dentists (*n* = 1)^47^ or nurses (*n* = 1).^41^ In all studies, the intervention duration was 3 min or less. One study took 2 to 3 min,^44^ 5 took 1 to 2 min,^39,40, 45, 46, 48^ 3 took about 1 min,^35,36, 41, 49^ and 3 took no more than 30 s.^37,38, 42, 43^ One study did not state the exact duration, but we regarded it as less than 3 min because the advice was described as only including tobacco harm on oral health and involved no intensive counseling by dentists.^47^

Ten studies standardized the intervention in an advice script (*n* = 6)^37,38, 41–43, 48, 49^ or guided by a clear protocol (*n* = 4)^35,36, 39, 44, 47^ and three studies mentioned that interventionists advised patients using their own style without a clearer intervention protocol.^40,45, 46^ In addition to verbal advice, 10 studies incorporated written materials such as leaflets and small cards.^35–38, 40, 41, 44–48^ One study delivered a “starter kit” including non-nicotine gums and rubber bands.^47^ Three studies included boosters of telephone follow-up after patients received the VBA.^38,41, 49^ One study included in-person clinic visit boosters.^44^

Eight studies included workshops or briefings about the intervention protocol to build capacity of VBA interventionists,^35,36, 38, 41–44, 47–49^ whereas three studies reported the workshops only took less than an hour.^38,42, 43, 48^ The remaining five studies did not report the VBA training for the interventionists.

### Risk of Bias

Six studies clearly mentioned the methods of randomization and concealment.^35–38, 41–43, 49^ No studies blinded participants or personnel about the intervention. Attrition bias was either low or unclear. Outcome reporting bias was mostly unclear or high. Considerable heterogeneity of methods was found specifically on selection bias and other bias. Details on assessing the risk of bias are shown in eResults 2.

We found no significant moderation by randomization generation, conflict of interest, and follow-up time points on the abstinence outcomes, supporting that an overall analysis integrating the available data was appropriate. Regarding quit attempts, we found that the heterogeneity of random sequence generation and operationalization of quit attempts moderated the quit attempt outcome; hence, three studies with high risk of bias were removed when pooling the risk ratio. Details on assessing the moderation effect of the study characteristics are shown in eResults 3.

### Effectiveness of Outcomes

**Figure 2:**
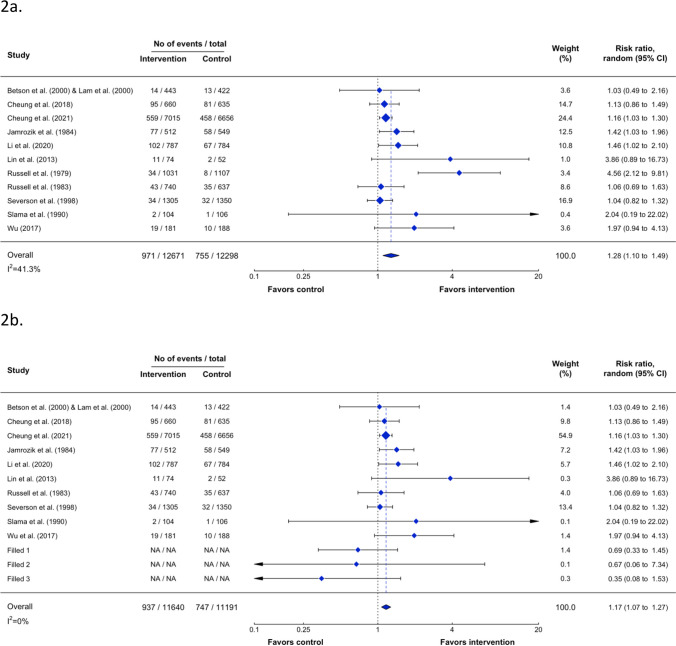
Forest plot of the average treatment affects tobacco abstinence assessed at ≥ 6 months before (**a**) and after (**b**) trim and fill analysis.

**Figure 3:**
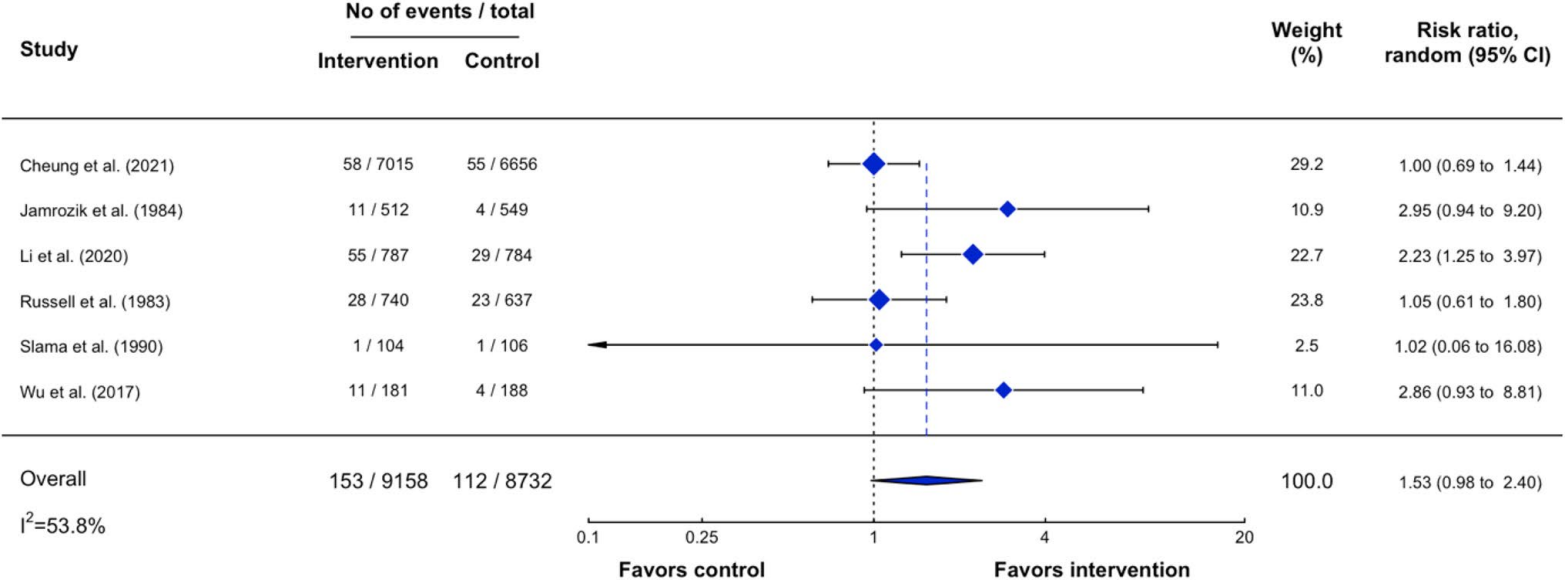
Forest plot of the average treatment effect on biochemically validated tobacco abstinence.

Publication bias was detected in abstinence assessed at ≥ 6 months and < 6 months. A trim and fill analysis was also conducted by imputing hypothetical studies to correct the funnel plot to be symmetrical (see eFigure 2a, b, 3a and b). The crude average treatment effect for abstinence assessed at ≥ 6 months was RR 1.28 (95% CI 1.10–1.49; NNT 66, 95%CI 46–112; Fig. 2a). After adjusting publication bias, the average treatment effect of VBA for tobacco abstinence at ≥ 6 months was small and significant (RR 1.17, 95% CI 1.07–1.27; NNT 73, 95%CI 49–143; Fig. 2b). The average treatment effect for biochemically validated abstinence from six studies was not significant, with a greater RR and wider confidence interval than self-reported abstinence (RR 1.53, 95% CI 0.98–2.40; NNT 256, 95%CI 135–2855; Fig. 3). The average treatment effect for abstinence at < 6 months was RR 1.35 (95% CI: 1.15–1.58; NNT 72, 95%CI 51–122; eFigure 4). After adjusting publication bias, the average treatment effect for tobacco abstinence at < 6 months was small and significant (RR 1.22, 95% CI: 1.01–1.47; NNT 82, 95%CI 55–166; in eFigure 5).

The average treatment effect from five studies for quit attempts was small and significant (RR 1.18, 95% CI: 1.02–1.35; eFigure 6). After excluding studies with high selection bias from pooled analysis, the average treatment effect for quit attempts was nearly null (RR 1.03, 95% CI: 0.97–1.08; eFigure 7).

Subgroup analysis on setting was not conducted because all included studies were done in clinical settings, except one study, which did not specify the setting. Also, the available studies did not report findings stratified by age subgroups. Analysis was only conducted on five studies with the subgroup aged 18 years and older. Moreover, only two studies with a low risk of bias reported quit attempts, thereby subgroup analysis was not done. Most subgroup analyses for age, economic status of countries, interventionists, control interventions, and length of advice showed significant intervention effects of VBA on abstinence outcomes at ≥ 6 months and < 6 months (eFigures 8–17). The intervention effect of VBA in low- and middle-income countries (i.e., China only, excluding Hong Kong) on abstinence at ≥ 6 months was moderate but not significant (eFigure 10: RR 1.57, 95%CI 0.89–2.78). When the advice length was 1–2 min, the effect was small and not significant (eFigure 14: RR 1.28, 95%CI 0.98–1.67).

### Certainty of Evidence

Our GRADE approach showed that the certainty of evidence for the treatment effect on abstinence at ≥ 6 months and < 6 months was moderate and high, respectively, but the evidence was of very low certainty for the treatment effect on quit attempts (eResults 4).

## DISCUSSION

The current review supplemented the review by Aveyard et al. (2012), which included very brief and brief interventions by including newer and more focused evidence to assess the effectiveness of VBA in 3 min or less. This world’s first meta-analysis on VBA showed that VBA, delivered in 3 min or less by healthcare professionals, effectively increased self-reported abstinence at ≥ 6 months by 17% and < 6 months by 22% compared to no SC advice, with moderate and high certainty of evidence respectively, but quit attempts showed very low certainty evidence.

The included studies showed variability in methodological rigor. For example, we found unclear random sequence generation and treatment concealment in a few studies. Our sensitivity analyses showed no significant differences in the treatment effect across studies with different methods. Also, integrating RCTs of lower quality in the meta-analysis did not lead to an inflation of the treatment effect size for increasing abstinence. Therefore, the variation in study methods did not compromise our conclusion on the effectiveness of VBA.

Our subgroup analyses on the treatment effect by age, economic status of countries, interventionists, and length of advice showed no significant moderation effect. The meta-analysis in high-income countries showed a significant treatment effect on increasing abstinence, whereas such an effect in low- or middle-income countries was not significant, probably because of the small sample size. Our analysis included only three studies in China, which is an upper middle-income country; hence, more RCTs on the effectiveness of VBA, especially in other low- or middle-income countries, are warranted.

The outcome of quit attempts could be extracted from only five studies. Most of the studies did not consistently define a meaningful quit attempt, as the length of abstinence varied from 24 h to 7 days. As the evidence had very low certainty, the findings should be interpreted cautiously. The true effect could in fact be higher, given that quit attempts are a pre-requisite of eventual abstinence. More precise measures and consistent, high-quality evidence are needed to show stronger certainty of the effectiveness of quit attempts.

We showed that VBA had a smaller effect size than brief intervention and medication. Hence, VBA should not replace or reduce the delivery of other effective behavioral interventions when the latter are feasible and available. If the settings can facilitate longer consultations with the patients or have sufficient capacity to deliver intensive treatment, VBA only may not optimize the quitting outcomes. Healthcare professionals need to evaluate all the contextual factors and incorporate appropriate service models for smoking cessation.

Our qualitative synthesis of different VBA implementation strategies highlighted a few features which can be included in future VBA guidelines. Firstly, in three studies, clinicians only needed brief training of about an hour or less before delivering the intervention.^35,36, 38, 41–44, 47–49^ Since healthcare professionals already have extensive knowledge about the harms of tobacco and benefits of quitting, future training should emphasize the effectiveness and operation of VBA. They can certainly save lives and prevent serious smoking-induced diseases by spending little time and effort; even most smokers would not succeed quickly. Secondly, clear and specific advice models (e.g., 2A1R model, ABC model, AWARD model) conceptualize what the key components in a VBA, and these models should help health professionals understand what the “must-do” advice is. Even if they are not familiar with these models, they can simply warn about the high mortality due to smoking, that one out of two smokers will be killed by smoking,^50–52^ advise to quit as soon as possible and refer. Last, other quitting support such as referral to SC services when available, printed resources, and follow-up boosters (e.g., phone calls) can be incorporated into VBA when some smokers want more quitting support, but the evidence of these additional efforts is unclear.

## LIMITATIONS

The study had several limitations. First, the variety of the selected RCTs was limited. There is lack of RCTs testing the effectiveness of VBA in community-based settings, such as community health centers and health promotional campaigns. Only two RCTs included nurses or dentists as interventionists. Most studies predominantly recruited cigarette smokers and very few included other tobacco products. Hence, our findings have limited generalizability in these areas. Second, treating self-reported smoking abstinence as primary outcome without biochemical validation is another limitation of this review. Over-reporting of quitting is possible when using self-report alone, which may lead to inaccurate effect size estimates and biased results if over-reporting is not evenly distributed between intervention and control groups. Third, we did not require the presence of SC services as a selection criterion, because the present study aimed to test the effectiveness of offering VBA, regardless of using SC services or support following the delivery of VBA. We found that four studies indicating the availability of local smoking cessation services.^38,41–43^ Such information in other studies was not reported, so we could not ascertain if these services were really not available, and subgroup analysis of this feature would not yield reliable results. Last, 10 out of the 13 included studies did not incorporate any validation of interventionist compliance with the intervention protocol. For advice intended to be delivered in just a few minutes without validating compliance, the effects measured might overestimate the true impact of the advice. Future research needs to incorporate objective mechanisms to assess whether very brief advice is adequately delivered by interventionists and determine the actual “dose” and duration of the intervention delivered.

## CONCLUSIONS

This study showed that VBA had a significant although with a small effect size in increasing abstinence assessed at ≥ 6 months or < 6 months after treatment initiation. Our finding supports a call of action on delivering VBA in all contacts to patients who smoke in clinical settings. The simplicity, low cost, and high reach level of VBA intervention supports a wider implementation to further increase tobacco abstinence.

## Supplementary Information

Below is the link to the electronic supplementary material.

Supplementary file1 (DOCX 2.23 MB)

## Data Availability

The data that support the findings of this study are available from the corresponding author upon reasonable request.
