# Supplementary material for: Effectiveness of Very Brief Advice on Tobacco Cessation: A Systematic Review and Meta-Analysis
Source: J Gen Intern Med. 2024 May 2;39(9):1721–34. doi: 10.1007/s11606-024-08786-8 (PMC11255176; doi:10.1007/s11606-024-08786-8)
Supplement: Supplementary file 1 — Supplementary file1 (DOCX 2.23 MB) [file 11606_2024_8786_MOESM1_ESM.docx]

**Supplemental contents**

**eMethods**. Database search strategy

**eResults 1** Study and participant characteristics

**eResults 2.** Methodological quality

**eFigure 1.** Stacked bar for risk of bias summary across all included studies

**eTable 1**. Risk of bias for each domain in each individual study

**eResults 3.** Heterogeneity of methodology

**eTable 2**. Moderation effects of study characteristics on the pooled estimates

**eFigure 2a.** Funnel Plot for the tobacco abstinence assessed at ≥6 months before trim and fill analysis

**eFigure 2b.** Funnel Plot for the long-term tobacco abstinence assessed at ≥6 months after trim and fill analysis

**eFigure 3a.** Funnel Plot for tobacco abstinence assessed at <6 months before trim and fill analysis

**eFigure 3b.** Funnel Plot for the tobacco abstinence assessed at <6 months after trim and fill analysis

**eFigure 4** – Forest Plot of the average treatment effect on tobacco abstinence assessed at <6 months before trim and fill analysis

**eFigure 5** – Forest Plot of the average treatment effect on tobacco abstinence assessed at <6 months after trim and fill analysis

**eFigure 6** – Forest Plot of the average treatment effect on quit attempts including all available studies

**eFigure 7** – Forest Plot of the average treatment effect on quit attempts excluding studies with high bias

**eFigure 8.** Forest Plot of the average treatment effect on tobacco abstinence assessed at ≥6 months from studies only including adults aged 18 year or above

**eFigure 9.** Forest Plot of the average treatment effect on tobacco abstinence assessed at <6 months from studies only including adults aged 18 year or above

**eFigure 10.** Forest Plot of the average treatment effect on tobacco abstinence assessed at ≥6 months from studies conducted in high-income versus low- and middle-income countries (economic status of countries), excluding Russell et al. 1979 and the other filled studies

**eFigure 11.** Forest Plot of the average treatment effect on tobacco abstinence assessed at <6 months from studies conducted in high-income versus low- and middle-income countries (economic status of countries), excluding Russell et al. 1979 and the other filled studies

**eFigure 12.** Forest Plot of the average treatment effect on tobacco abstinence assessed at ≥6 months from studies of different interventionists subgroup, excluding Russell et al. 1979 and the other filled studies

**eFigure 13.** Forest Plot of the average treatment effects on tobacco abstinence assessed at <6 months from studies of different interventionists subgroup, excluding Russell et al. 1979 and the other filled studies

**eFigure 14.** Forest Plot of the average treatment effect on tobacco abstinence assessed at ≥6 months from studies of different length of advice

**eFigure 15.** Forest Plot of the average treatment effects on tobacco abstinence assessed at <6 months from studies of different length of advice

**eResults 4.** Certainty of evidence

**eTable 3** Summary of findings by GRADE approach

**eMethods**. Database search strategy

A. English Database for quitting cigarette

1. Search strategy for MEDLINE (via OVID)

ID Subject search terms

1 exp smoker/ OR smoker.af.

ID Very Brief Advice search terms

2 (brief or simple).af.

3 (advice or intervention or treatment or counselling).af.

4 2 AND 3

ID Control group search terms

5 (usual or clinic* or standard* or routine or convention* or gener* or manual* or manag*).af.

ID Study domain search terms

6 Smoking.af. OR Tobacco.af. OR (Tobacco smoking).af. OR Cigarette.af.

ID outcome search terms

7 exp smoking cessation/

8 smoking reduc*.af.

9 (cut* down or cut-down).af.

10 (reduc* or stop* or quit* or abstin* or abstain* or cessat*).af.

11 smoking cessat*.af.

12 fading.af.

13 taper.af.

14 controlled smoking.af.

15 7 OR 8 OR 9 OR 10 OR 11 OR 12 OR 13 OR 14

ID RCT search terms

16 ((clin$ adj5 trial$) or random$).af.

17 1 and 4 and 5 and 6 and 15 and 16

2. Search strategy for CINAHL Plus (via EBSCOhost)

smoker AND (brief or simple) AND (advice or intervention or treatment or counselling) AND (usual or clinic* or standard* or routine or convention* or gener* or manual* or manag*) AND (MH "Smoking+") OR (MH "Tobacco+") AND (MH "Smoking Cessation") OR (MH "Smoking Cessation Programs") OR (MH "Tobacco Use Cessation Products") OR (MH "Smoking Cessation Assistance (Iowa NIC)") OR (MH "Smoke Inhalation Injury") OR (MH "Smoke Alarms") OR (MH "Passive Smoking") OR (MH "Smoke, Surgical") OR (MH "Smoke Evacuation") OR (MH "Smoking+") OR (MH "Abuse Cessation (Iowa NOC)") OR (MH "Smoke+") OR (MH "Treatment Withdrawal") OR (MH "Substance Abstinence+") AND (smoking reduc* or cut* down or cut-down or reduc* or stop* or quit* or abstin* or abstain* or cessat* or smoking cessat* or fading OR taper OR controlled smoking) AND (random* OR control*)

3. Search strategy for Embase (via OVID)

ID Subject search terms

1 exp smoker/ OR smoker.af.

ID Very Brief Advice search terms

2 (brief or simple).af.

3 (advice or intervention or treatment or counselling).af.

4 2 AND 3

ID Control group search terms

5 (usual or clinic* or standard* or routine or convention* or gener* or manual* or manag*).af.

ID Study domain search terms

6 Smoking.af. OR Tobacco.af. OR (Tobacco smoking).af. OR Cigarette.af.

ID outcome search terms

7 exp smoking cessation/

8 smoking reduc*.af.

9 (cut* down or cut-down).af.

10 (reduc* or stop* or quit* or abstin* or abstain* or cessat*).af.

11 smoking cessat*.af.

12 fading.af.

13 taper.af.

14 controlled smoking.af.

15 7 OR 8 OR 9 OR 10 OR 11 OR 12 OR 13 OR 14

ID RCT search terms

16 ((clin$ adj5 trial$) or random$ ).af.

17 1 and 4 and 5 and 6 and 15 and 16

4. Search strategy for APA PsycInfo (via ProQuest)

noft(Smoker) AND noft((brief OR simple)) AND noft((advice OR intervention OR treatment OR counselling)) AND noft((usual OR clinic* OR standard* OR routine OR convention* OR gener* OR manual* OR manag*)) AND noft((Smoking OR Tobacco OR Tobacco smoking OR Cigarette)) AND (MAINSUBJECT.EXACT.EXPLODE("Smoking Cessation") OR noft(smoking reduc* or cut* down or cut-down or reduc* or stop* or quit* or abstin* or abstain* or cessat* or smoking cessat* or fading OR taper OR controlled smoking)) AND noft((random* OR control*))

5. Search strategy for Cochrane library

#1 (random*):ti,ab,kw

#2 MeSH descriptor: [Smokers] explode all trees

#3 (brief or simple):ti,ab,kw

#4 (advice or intervention or treatment or counselling):ti,ab,kw

#5 #2 AND #3 AND #4

#6 (usual or clinic* or standard* or routine or convention* or gener* or manual* or manag*):ti,ab,kw

#7 Smoking OR Tobacco OR Tobacco smoking OR Cigarette

#8 MeSH descriptor: [smoking cessation] explode all trees

#9 (smoking reduc*):ti,ab,kw

#10 (cut* down or cut-down):ti,ab,kw

#11 (reduc* or stop* or quit* or abstin* or abstain* or cessat*):ti,ab,kw

#12 (smoking cessat*):ti,ab,kw

#13 (fading):ti,ab,kw

#14 (taper):ti,ab,kw

#15 (controlled smoking):ti,ab,kw

#16 #8 OR #9 OR #10 OR #11 OR #12 OR #13 OR #14 OR #15

#17 #1 and #5 and #6 and #7 and #16

6. Search strategy for ClinicalTrials.gov and WHO-ICTRP

Smoker AND (brief OR simple) AND (advice OR intervention OR treatment OR counselling) AND (Smoking OR Tobacco OR Tobacco smoking OR Cigarette) | Smoking

B. English Database Search for quitting other tobacco products

1. Search strategy for MEDLINE (via OVID)

ID Subject search terms

1 exp smoker/ OR smoker.af.

ID Very Brief Advice search terms

2 (brief or simple).af.

3 (advice or intervention or treatment or counselling).af.

4 2 AND 3

ID Control group search terms

5 (usual or clinic* or standard* or routine or convention* or gener* or manual* or manag*).af.

ID Study domain search terms

6 Cigar.af. OR shisha.af. OR hookah.af. OR snus.af. OR snuff.af. OR chewing tobacco.af. OR dip.af. OR smokeless tobacco.af. OR heat-not-burn.af. OR heat not burn.af.

ID outcome search terms

7 exp smoking cessation/

8 smoking reduc*.af.

9 (cut* down or cut-down).af.

10 (reduc* or stop* or quit* or abstin* or abstain* or cessat*).af.

11 smoking cessat*.af.

12 fading.af.

13 taper.af.

14 controlled smoking.af.

15 7 OR 8 OR 9 OR 10 OR 11 OR 12 OR 13 OR 14

ID RCT search terms

16 ((clin$ adj5 trial$) or random$).af.

17 1 and 4 and 5 and 6 and 15 and 16

2. Search strategy for CINAHL Plus (via EBSCOhost)

smoker AND (brief or simple) AND (advice or intervention or treatment or counselling) AND (usual or clinic* or standard* or routine or convention* or gener* or manual* or manag*) AND (MH “Cigar”) OR (MH “shisha”) OR (MH “hookah”) OR (MH “snus”) OR (MH “snuff”) OR (MH “chewing tobacco”) OR (MH “dip”) OR (MH “smokeless tobacco”) OR (MH ”heat-not-burn”) OR (MH ”heat not burn”) AND ((MH "Smoking Cessation") OR (MH "Smoking Cessation Programs") OR (MH "Tobacco Use Cessation Products") OR (MH "Smoking Cessation Assistance (Iowa NIC)") OR (MH "Smoke Inhalation Injury") OR (MH "Smoke Alarms") OR (MH "Passive Smoking") OR (MH "Smoke, Surgical") OR (MH "Smoke Evacuation") OR (MH "Smoking+") OR (MH "Abuse Cessation (Iowa NOC)") OR (MH "Smoke+") OR (MH "Treatment Withdrawal") OR (MH "Substance Abstinence+")) AND (smoking reduc* or cut* down or cut-down or reduc* or stop* or quit* or abstin* or abstain* or cessat* or smoking cessat* or fading OR taper OR controlled smoking) AND (random* OR control*)

3. Search strategy for Embase (via OVID)

ID Subject search terms

1 exp smoker/ OR smoker.af.

ID Very Brief Advice search terms

2 (brief or simple).af.

3 (advice or intervention or treatment or counselling).af.

4 2 AND 3

ID Control group search terms

5 (usual or clinic* or standard* or routine or convention* or gener* or manual* or manag*).af.

ID Study domain search terms

6 Cigar.af. OR shisha.af. OR hookah.af. OR snus.af. OR snuff.af. OR chewing tobacco.af. OR dip.af. OR smokeless tobacco.af. OR heat-not-burn.af. OR heat not burn.af. OR e-cigar*.af.

ID outcome search terms

7 exp smoking cessation/

8 smoking reduc*.af.

9 (cut* down or cut-down).af.

10 (reduc* or stop* or quit* or abstin* or abstain* or cessat*).af.

11 smoking cessat*.af.

12 fading.af.

13 taper.af.

14 controlled smoking.af.

15 7 OR 8 OR 9 OR 10 OR 11 OR 12 OR 13 OR 14

ID RCT search terms

16 ((clin$ adj5 trial$) or random$ ).af.

17 1 and 4 and 5 and 6 and 15 and 16

4. Search strategy for APA PsycInfo (via ProQuest)

noft(Smoker) AND noft((brief OR simple)) AND noft((advice OR intervention OR treatment OR counselling)) AND noft((usual OR clinic* OR standard* OR routine OR convention* OR gener* OR manual* OR manag*)) AND noft(Cigar OR shisha OR hookah OR snus OR snuff OR chewing tobacco OR dip OR smokeless tobacco OR heat-not-burn OR heat not burn AND (MAINSUBJECT.EXACT.EXPLODE("Smoking Cessation") OR noft(smoking reduc* or cut* down or cut-down or reduc* or stop* or quit* or abstin* or abstain* or cessat* or smoking cessat* or fading OR taper OR controlled smoking)) AND noft((random* OR control*))

5. Search strategy for Cochrane library

#1 (random*):ti,ab,kw

#2 MeSH descriptor: [Smokers] explode all trees

#3 (brief or simple):ti,ab,kw

#4 (advice or intervention or treatment or counselling):ti,ab,kw

#5 #2 AND #3 AND #4

#6 (usual or clinic* or standard* or routine or convention* or gener* or manual* or manag*):ti,ab,kw

#7 (Cigar OR shisha OR hookah OR snus OR snuff OR chewing tobacco OR dip OR smokeless tobacco OR heat-not-burn OR heat not burn

#8 MeSH descriptor: [smoking cessation] explode all trees

#9 (smoking reduc*):ti,ab,kw

#10 (cut* down or cut-down):ti,ab,kw

#11 (reduc* or stop* or quit* or abstin* or abstain* or cessat*):ti,ab,kw

#12 (smoking cessat*):ti,ab,kw

#13 (fading):ti,ab,kw

# bnbn #16 #8 OR #9 OR #10 OR #11 OR #12 OR #13 OR #14 OR #15

#17 #1 and #5 and #6 and #7 and #16

6. Search strategy for ClinicalTrials.gov and WHO-ICTRP

Smoker AND (brief OR simple) AND (advice OR intervention OR treatment OR counselling) AND (Cigar OR shisha OR hookah OR snus OR snuff OR chewing tobacco OR dip OR smokeless tobacco OR heat-not-burn OR heat not burn

C. Chinese database search strategy for quitting cigarette and other tobacco products

1. Search strategy for Wanfang(万方), Chaoxing Periodicals (Superstar 超星期刊) and China National Knowledge Infrastructure (CNKI) (中国知识网络服务平台)

(简短干预 OR 精简干预) AND 戒烟

2. Search strategy for Airiti Library (華藝線上圖書館), Taiwan Periodical Literature (台灣期刊論文) and Government Research Bulletin (政府研究資訊醫療照護系統)

(簡短干預 OR 精簡干預) AND 戒菸

**eResults 1.** Study and participant characteristics

A total of 13 RCTs (no cluster RCTs) from 15 articles (n=26,437) published from 1979 to 2021 were included in the synthesis [35,36,37,38,39,40,41,42,43,44,45,46,47,48,49] (Figure 1). The extracted general information and abstinence results in each study are presented in Table 1 and Table 2, respectively. Nine RCTs were from high income countries/regions (United Kingdom, United States, Hong Kong, Australia, and Canada) [35,36,37,39,40,41,45,46,47,48] and 4 from China, an upper middle-income country [38,42,43,44,49]. The trials results from Hong Kong in 1994-1995 [35,36] and in Guangzhou China in 2009-2010 (43, 44) were both found or published in two articles. Twelve studies recruited subjects in outpatient clinics (n=10) [35,36,38,39,40,42,43,44,45,46,47,49] and emergency departments (n=2) [37,41](38, 42), and 1 study did not specify the setting (49). All studies recruited predominantly cigarette users only [35,36,37,38,39,40,41,42,43,44,45,46,47,48,49] and only one included a small number of exclusive cigar or unspecified tobacco users (n=40)[37].

Six studies included subjects aged 18 years or above [37,38,41,44,48,39], 3 included subjects aged 16 years or above [39,45,46], 2 included subjects aged 15 years or above [35,36,47], and 2 did not specify age criteria [39,42,43]. Interventions were mainly delivered by physicians (n=11) [35,36,37,38,39,40,42,43,44,45,46,48,49] and the others by dentists (n=1) [47] or nurses (n=1) [41]. In all studies, the intervention duration was 3 minutes or less. One study took 2 to 3 minutes [44], 5 took 1 to 2 minutes [39,40,45,46,48], 3 took about 1 minute [35,36,41,49], and 3 took no more than 30 seconds [37,38,42,43]. One study did not state the exact duration but we regarded it as less than 3 minutes because the advice was described as only including tobacco harm on oral health and involved no intensive counselling by dentists [47].

Ten studies standardized the intervention in an advice script (n=6) [37,38,41,42,43,48,49] or guided by a clear protocol (n=4) [35,36,39,44,47] and 3 studies mentioned that interventionists advised patients using their own style without a clearer intervention protocol [40,45,46]. In addition to verbal advice, 10 studies incorporated written materials such as leaflets and small cards [35,36,37,38,40,41,44,45,46,47,48]. One study delivered a “starter kit” including non-nicotine gums and rubber bands [47]. Three studies included boosters of telephone follow-up after patients received the VBA [38,41,49]. One study included in-person clinic visit boosters [44].

Eight studies included workshops or briefings about the intervention protocol to build capacity of VBA interventionists [35,36,38,41,42,43,44,47,48,49], where three studies reported the workshops only took less than an hour [38,42,43,48]. The remaining 5 studies did not report the VBA training for the interventionists.

Eleven studies (n=11) [35,36,37,38,40,41,42,43,45,46,47,48,49] assessed tobacco abstinence at 12 months after receiving the intervention. The remaining 2 studies assessed the quitting outcomes at time points sooner than 6 months after study enrolment [39,44]. Of studies with 1-year follow-up, various definitions of abstinence were used, including point-prevalence abstinence (n=3) [37,38,41], sustained abstinence for at least 6 months (n=7) [35,36,42,43,45,46,47,48,49], or unspecified length of abstinence (n=1) [40]. Six studies [38,40,41,45,46,47,48,49] included biochemical validated abstinence.

In the control arms of the 8 studies, participants generally received usual care with no specific SC advice or contact [37,39,40,42,43,44,45,46,47,48]. In one study, participants received a SC leaflet and 1-minute placebo booster calls during follow-ups that promote physical activity, fruit, and vegetable intake [41]. Another study involved 30-second advice about fruit and vegetable consumption, along with a leaflet and card [38]. Similarly, another study provided 1-minute exercise and diet advice, along with 1-minute booster calls on exercise and diet advice [49]. In one study, some participants received a SC booklet, while others had no intervention [35,36].

**eResults 2.** Methodological quality

*Selection bias*

Six studies had low risk of bias, which described the use of appropriated randomized technique [35,36,37,38,41,42,43,49]. Three studies had high risk of bias as randomization was done using the day (n=2) [40,45] or week (n=1) [46] of attendance. Four studies did not describe the randomization procedures. Six studies implemented allocation concealment (e.g. sequentially numbered, opaque and sealed envelope) but the other 7 studies had unclear concealment.

*Blinding*

Eight studies blinded the outcome assessors (n=8) [35,36,37,38,39,41,42,43,48,49] and the other 5 studies did not report sufficient information about the blinding of the outcome assessors (n=5) [40,44,45,46,47]. All studies did not blind the participants, practitioners and recruitment personnel about the intervention (n=13).

*Attrition bias*

Eight studies were judged as low risk of attrition bias since there are balanced number of missing outcome data across trial groups and similar reasons of missingness across trial groups [37,38,40,41,42,43,44,46,49]. The other 5 studies had unclear attrition bias [35,36,39,45,47,48] as limited information about the characteristics of the subjects was reported.

*Outcome Reporting bias*

Eight studies did not have a registered study protocol to include enough information for assessing the reporting bias [35,36,37,42,43,44,45,46,48,49]. Two had low risk of reporting bias as all study outcomes were reported in the pre-specified protocols [38,41]. Three studies had high risk of reporting bias as the outcome for quit attempts could not be assessed [39,40,47].

*Other bias*

Nine studies had high risk of bias due to undeclared conflict of interest [35,36,39,40,42,43,44,45,46,47,48]. Two studies had unclear risk of bias, of which a study contained only 1% female participants [38] and another one had inconsistency on the inclusion criteria other than outcomes between the protocol and report (i.e., “daily smokers” mentioned in the protocol, but “current smokers” was mentioned in the report) [41].

*Overall result*

Six studies clearly mentioned the method of randomization and concealment [35,36,37,38,41,42,43,49]. No studies blinded participants or personnel about the intervention. Attrition bias was either low or unclear. Outcome reporting bias was mostly unclear or high. Considerable heterogeneity of methodology was found specifically on selection bias and other bias. The moderation effect of the heterogeneity of random sequence generation on the average treatment effects was tested. Nine studies had not declared conflict of interest, the test for moderation effect of the heterogeneity of other risk bias on the average treatment effects was tested.

eFigure 1. Stacked bar for risk of bias summary across all included studies


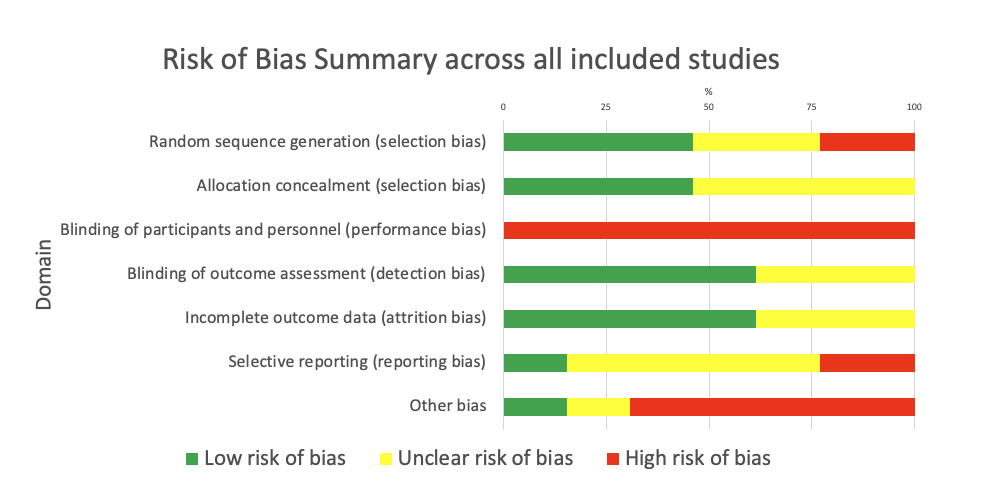


eTable 1. Risk of bias for each domain in each individual study


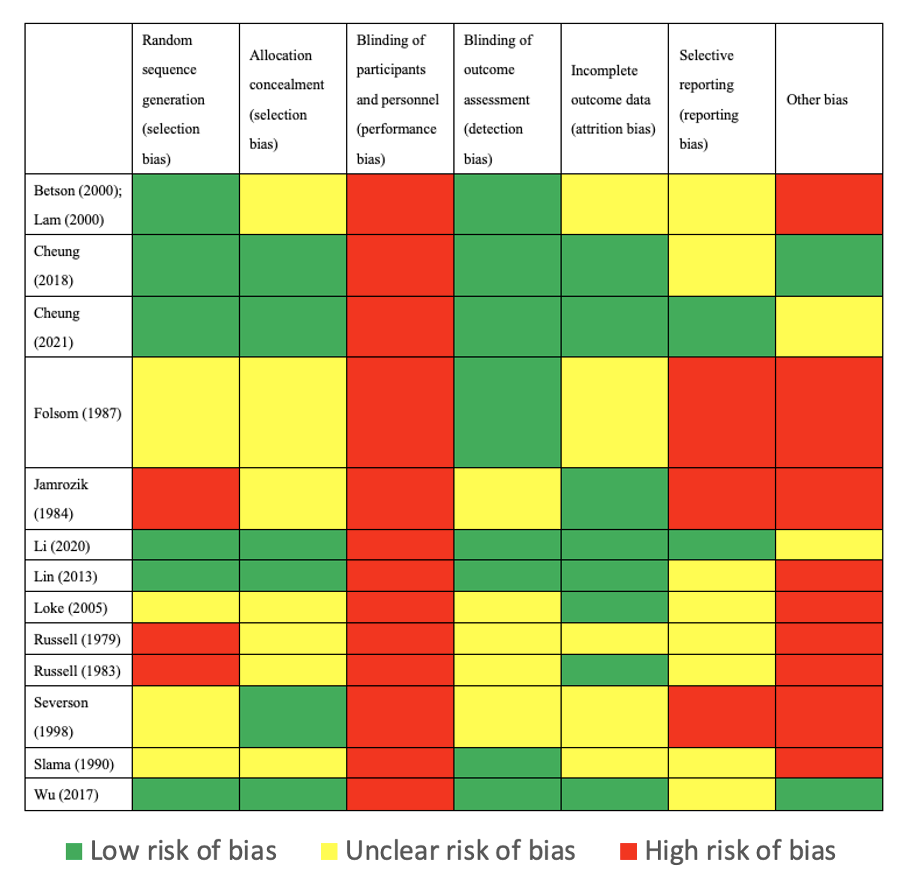


**eResults 3.** Heterogeneity of methodology

*Outcome of the studies*

Eleven studies (n=11) [35,36,37,38,40,41,42,43,45,46,47,48,49] assessed tobacco abstinence at 12 months after receiving the intervention. The remaining 2 studies assessed the quitting outcomes at time points sooner than 6 months after study enrolment [39,44]. Of studies with 1-year follow-up, various definitions of abstinence were used, including point-prevalent abstinence (n=3) [37,38,41], sustained abstinence for at least 6 months (n=7) [35,36,42,43,45,46,47,48,49], or unspecified length of abstinence (n=1) [40]. Specifically, we examined whether type of abstinence definition (point-prevalent vs. sustained vs. unspecified) significantly moderated the intervention effect in the meta-analysis. Six studies [38,40,41,45,46,47,48,49] included biochemical validated abstinence.

Seven studies [35,36,37,38,39,42,43,47,49] reported abstinence assessed at 3-month follow-up and were combined for meta-analysis. Other assessment timepoints included 1-month (n=2) [45,48], 4-month (n=1) [46], 3- to 5-month (n=1) [44]. Because of the differences in follow-up time points, we assessed the moderation effects before combining the studies. By grouping 4-month and 3- to 5-month into a group, we examined whether follow-up timepoints (1-month versus 3-month versus 3- to 5-month) significantly affected the estimated short-term abstinence rate.

Quit attempts were assessed in 8 studies [37,38,39,40,44,45,46,47], but 3 studies had incomplete data for our analysis [39,40,47]. In the other 5 studies, 1 study assessed quit attempts at 4 month [46], 1 assessed between 3 and 5 month [44] and 3 assessed at 12 month [37,38,45]. However, 3 of these 5 studies did not state clearly how to define quit attempts [44,45,46]; In the remaining two studies, 1 operationalized quit attempts as abstinence for at least 7 days [37] and another one operationalized it as abstinence for at least 24 hours [38]. Because of the differences in operationalizing quit attempt, we assessed the moderation effects whether the study with and without operationalization significantly affected the estimated treatment effect for quit attempts.

*Control interventions characteristics*

In the control arms of the 8 studies, participants generally received usual care with no specific SC advice or contact [37,39,40,44,45,46,47,48]. In one study, participants received a SC leaflet and 1-minute placebo booster calls during follow-ups that promote physical activity, fruit, and vegetable intake [41]. Another study involved 30-second advice about fruit and vegetable consumption, along with a relevant leaflet and card [38]. Similarly, another study provided 1-minute exercise and diet advice, along with 1-minute booster calls on exercise and diet advice [49]. In one study, some participants received a SC booklet, while others had no intervention [35,36].

*Comparability of studies*

The moderation effect of study characteristics on pooled estimate could be found on eTable 2. The moderation for the studies with bias of random sequence generation (high versus low/unclear) on abstinence ≥6 months (Chi² = .42, p = .52) and <6 months (Chi² = .36, p = .55) were insignificant. Also, our findings did not support that types of abstinence (point-prevalent, sustained, or unspecified) moderated the intervention effect of VBA on abstinence ≥6 months (Chi² = 1.59, p = .45). The test for moderation by follow-up time points on abstinence <6 months indicated no evidence of moderation effect (Chi² = .93, p = .63). Moderation effect of presence of conflict of interest on abstinence ≥6 months (Chi² = .068, p = .79) and <6 months (Chi² = .50, p = .48) were insignificant. To conclude, there was no evidence that the heterogeneity of methods moderated the abstinence outcomes, suggesting an overall analysis integrating the available data was suitable.

The moderation for the studies with bias of random sequence generation (high versus low/unclear) on quit attempts was significant (Chi^2^ = 13.97, p < .001). However, there was significant moderation due to operationalization on quit attempts (with clear operationalization versus without a clear operationalization) (Chi² = 18.62, p < .001). Coincidently, the studies without a clear operationalization for quit attempt also did not declare conflict of interest, therefore the bias due to difference in conflict of interest on quit attempts was significant (Chi² = 18.62, p < .001). Regarding to quit attempts, since the heterogeneity of study characteristics moderated the quit attempts, those studies with high risk of bias were removed in pooling the risk ratio.

eTable 2. Moderation effects of study characteristics on the pooled estimates

| Moderation effects | Chi^2^ | p-value |
| --- | --- | --- |
| Presence of Random Sequence Generation |  |  |
| Abstinence at ≥6 months | .42 | .52 |
| Abstinence at <6 months | .36 | .55 |
| Quit attempts | 13.97 | <.001 |
|  |  |  |
| Presence of Conflict of interest |  |  |
| Abstinence at ≥6 months | .068 | .79 |
| Abstinence at <6 months | .50 | .48 |
| Quit attempts | 18.62 | <.001 |
|  |  |  |
| Definition of abstinence in studies with abstinence at ≥6 months | 1.59 | .45 |
|  |  |  |
| Follow-up time points in studies with abstinence at<6 months | .93 | .63 |
|  |  |  |
| Types of definition on quit attempts | 18.62 | <.001 |

eFigure 2a. Funnel Plot for the tobacco abstinence assessed at ≥6 months before trim and fill analysis


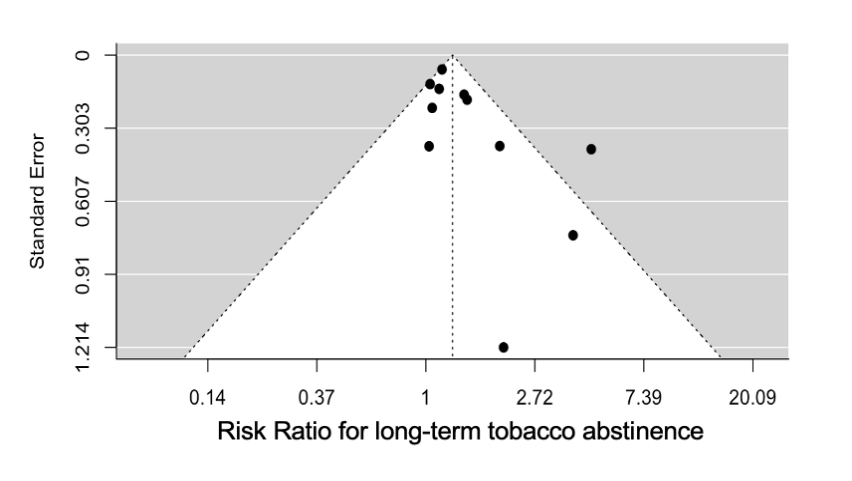


eFigure 2b. Funnel Plot for the long-term tobacco abstinence assessed at ≥6 months after trim and fill analysis


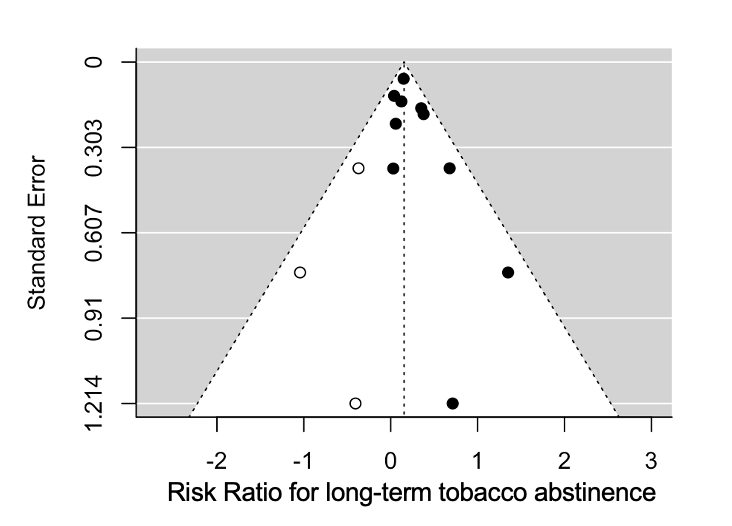


For abstinence assessed at ≥6 months, Russell et al.’s (45) was significantly sensitive to the result from the test of funnel plot asymmetry (before: Z = 2.65, P = .008; after: Z = 1.60, P = .11). We removed Russell et al. (45) and imputed 3 hypothetical missing studies to correct the funnel plot to be symmetrical.

eFigure 3a. Funnel Plot for tobacco abstinence assessed at <6 months before trim and fill analysis


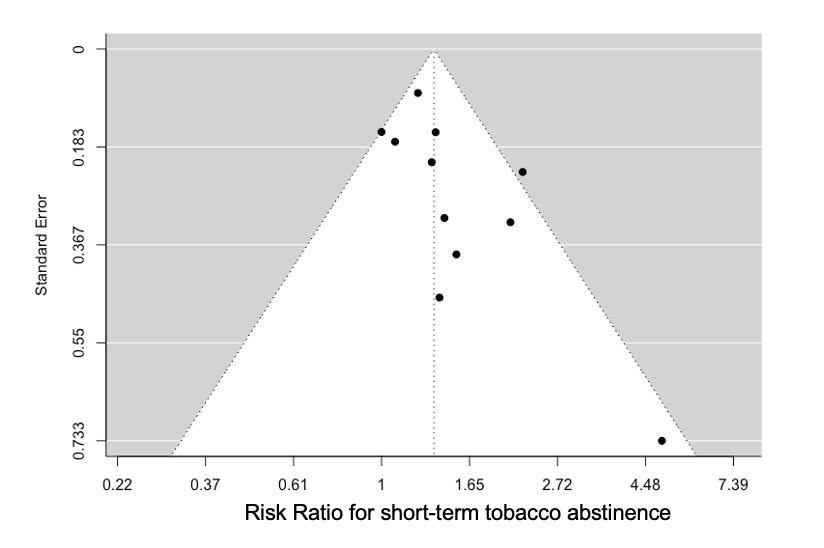


eFigure 3b. Funnel Plot for the tobacco abstinence assessed at <6 months after trim and fill analysis


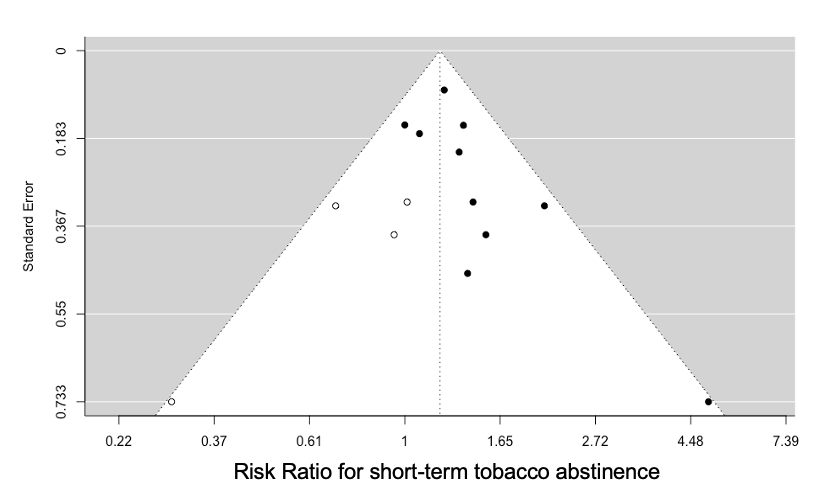


For abstinence assessed at <6 months, Russell et al. [45] was also significantly sensitive to the test of funnel plot asymmetry (before: Z = 2.22, P = .026; after Z = .089, P = .37). We removed Russell et al. [45] and imputed 4 hypothetical missing studies to correct the funnel plot to be symmetrical.

eFigure 4. Forest Plot of the average treatment effect on tobacco abstinence assessed at <6 months before trim and fill analysis


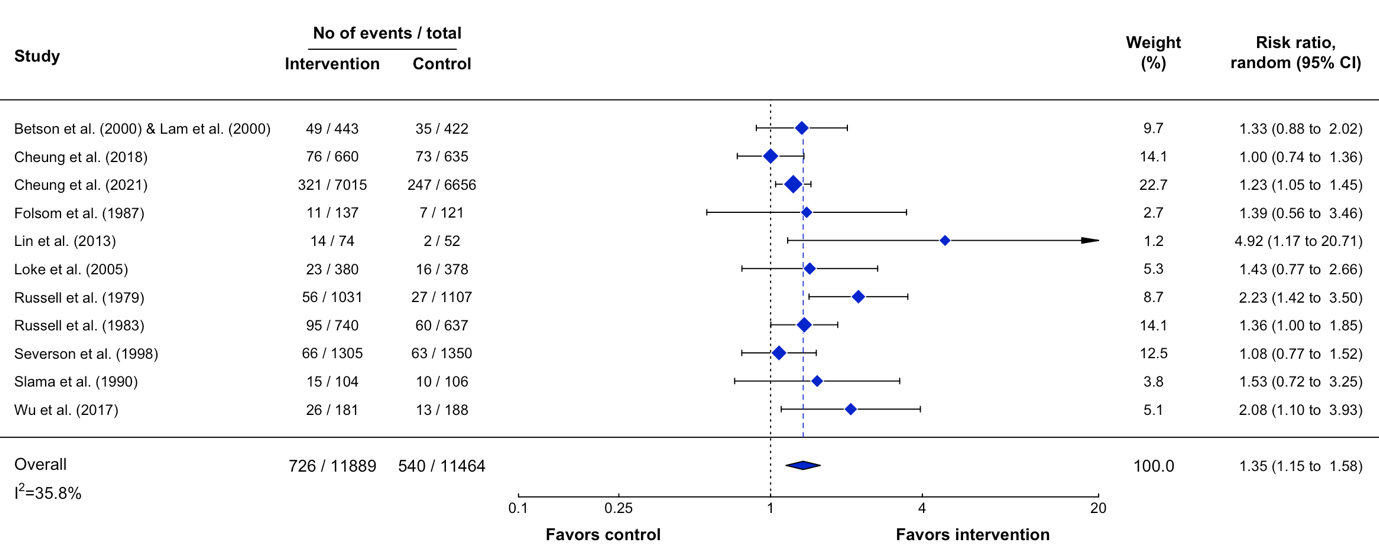


eFigure 5. Forest Plot of the average treatment effect on tobacco abstinence assessed at <6 months after trim and fill analysis


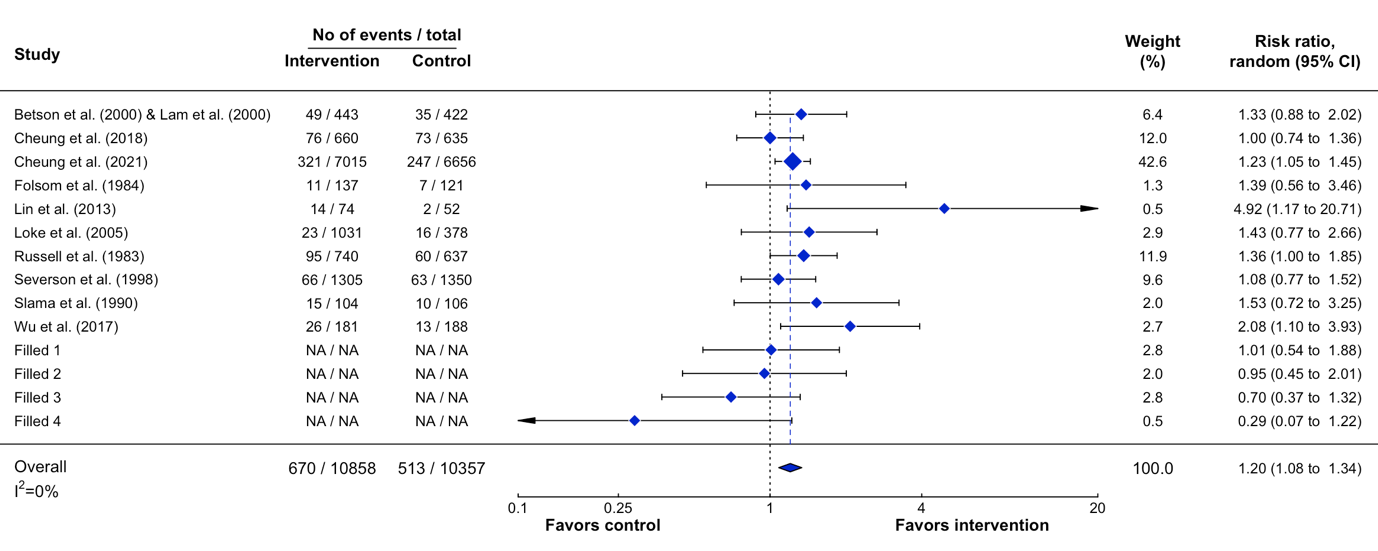


eFigure 6. Forest Plot of the average treatment effect on quit attempts including all available studies


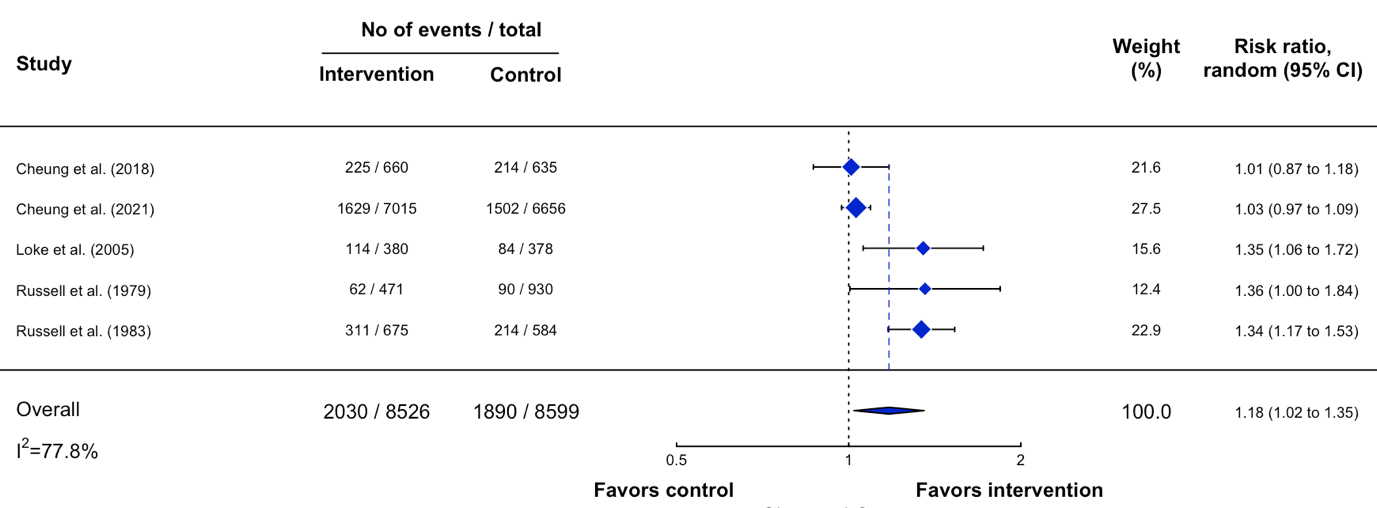


eFigure 7. Forest Plot of the average treatment effect on quit attempts excluding studies with high bias

*
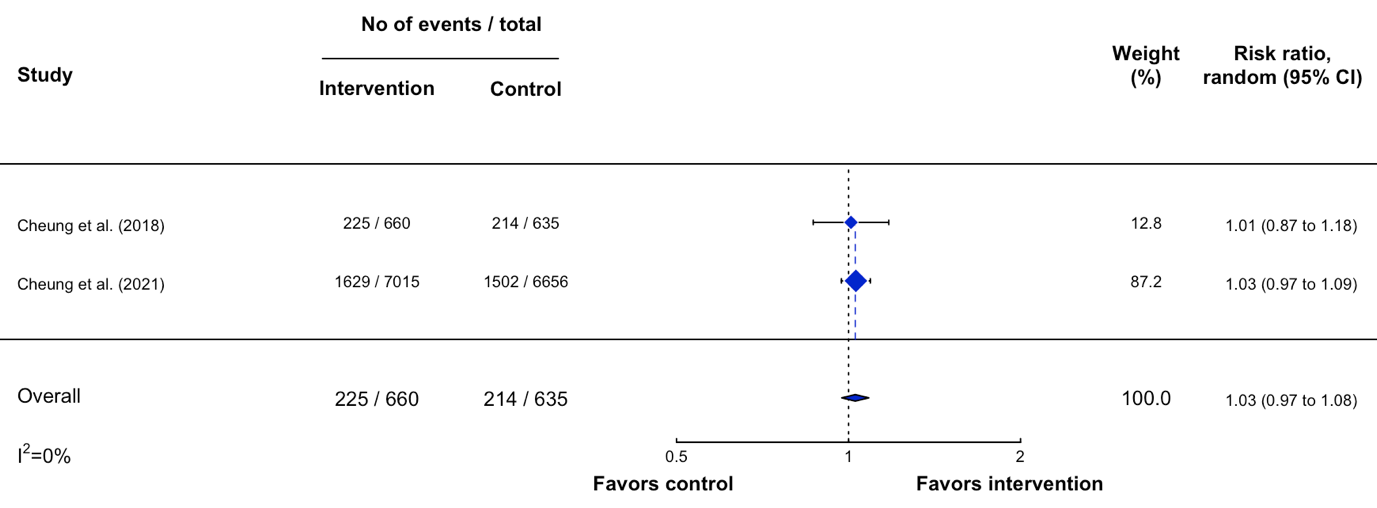
*

eFigure 8. Forest Plot of the average treatment effect on tobacco abstinence assessed at ≥6 months from studies only including adults aged 18 year or above
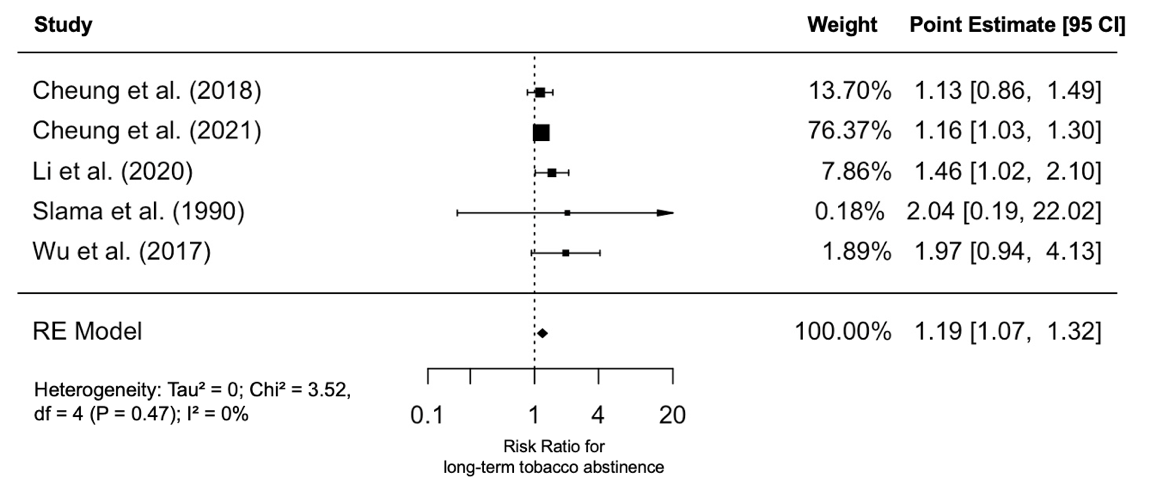


eFigure 9. Forest Plot of the average treatment effect on tobacco abstinence assessed at <6 months from studies only including adults aged 18 year or above
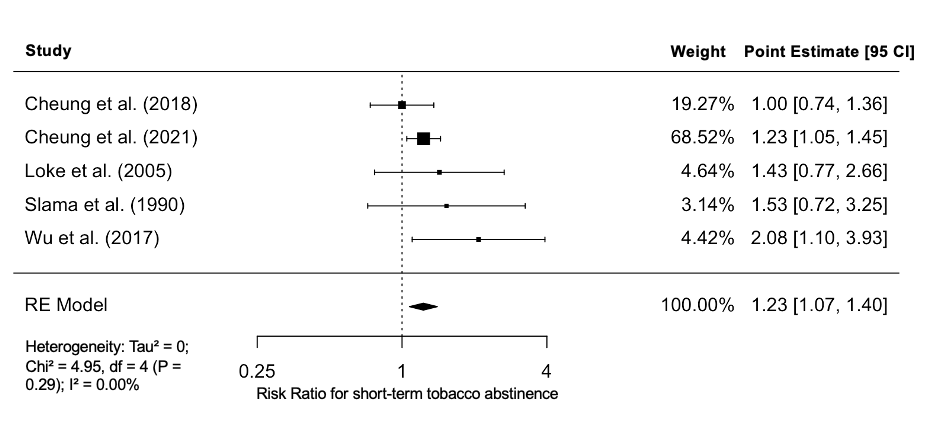


eFigure 10. Forest Plot of the average treatment effect on tobacco abstinence assessed at ≥6 months from studies conducted in high-income versus low- and middle-income countries (economic status of countries), excluding Russell et al. 1979 and the other filled studies
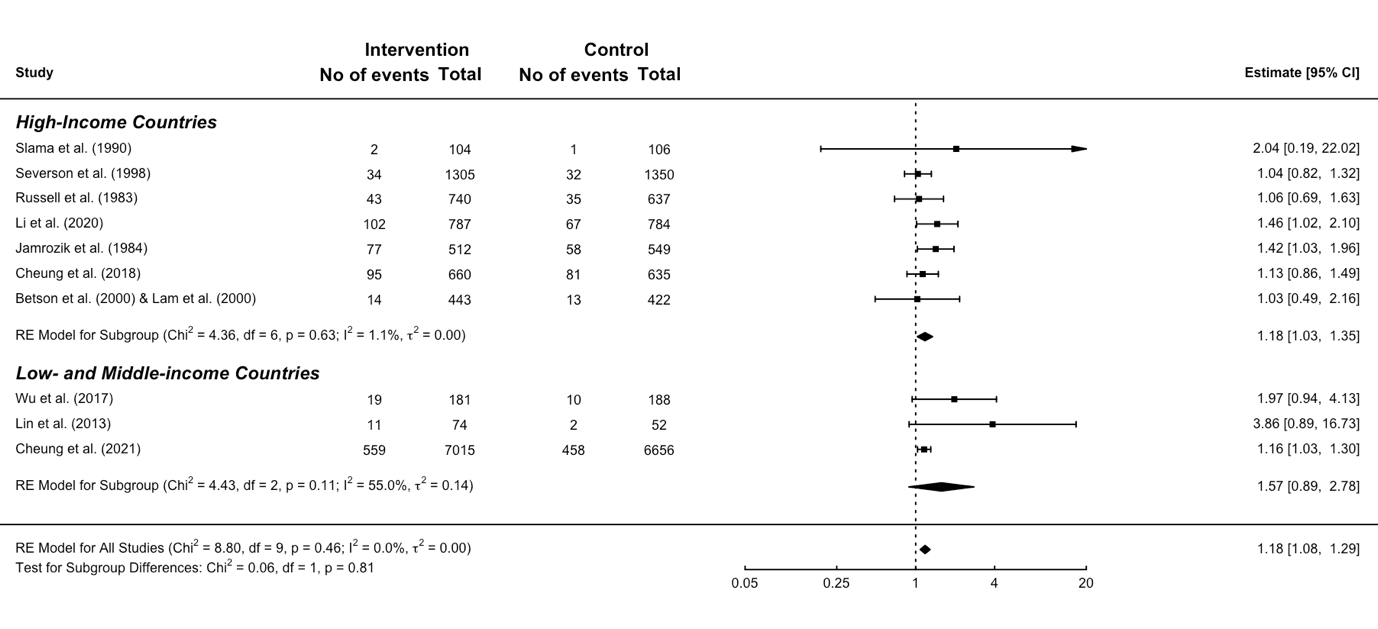


eFigure 11. Forest Plot of the average treatment effect on tobacco abstinence assessed at <6 months from studies conducted in high-income versus low- and middle-income countries (economic status of countries), excluding Russell et al. 1979 and the other filled studies
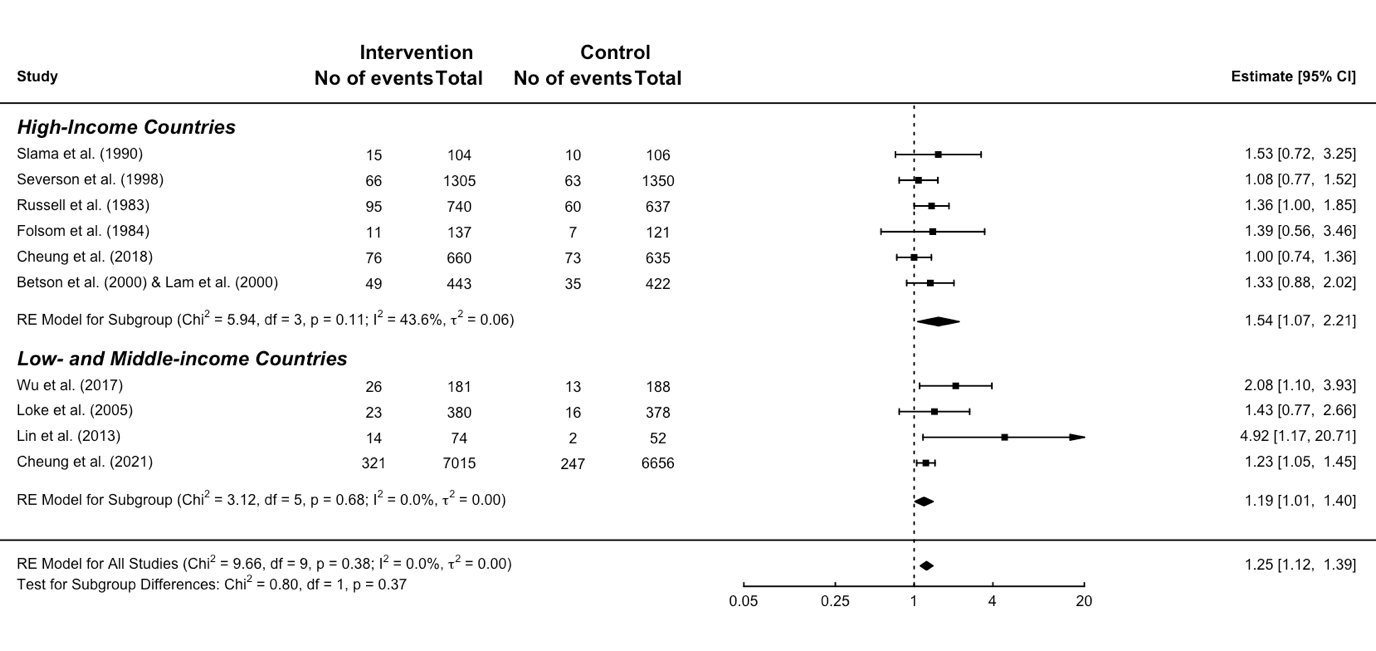


eFigure 12. Forest Plot of the average treatment effect on tobacco abstinence assessed at ≥6 months from studies of different interventionists subgroup, excluding Russell et al. 1979 and the other filled studies


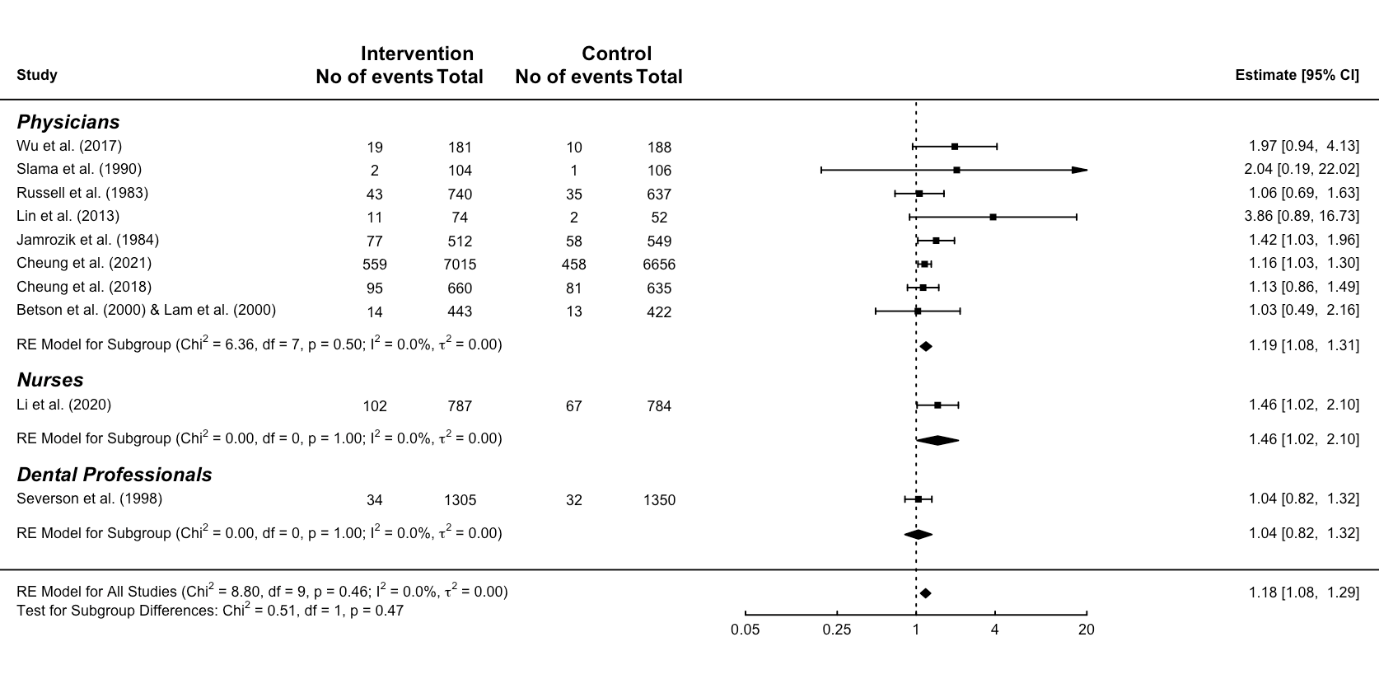


eFigure 13. Forest Plot of the average treatment effects on tobacco abstinence assessed at <6 months from studies of different interventionists subgroup, excluding Russell et al. 1979 and the other filled studies
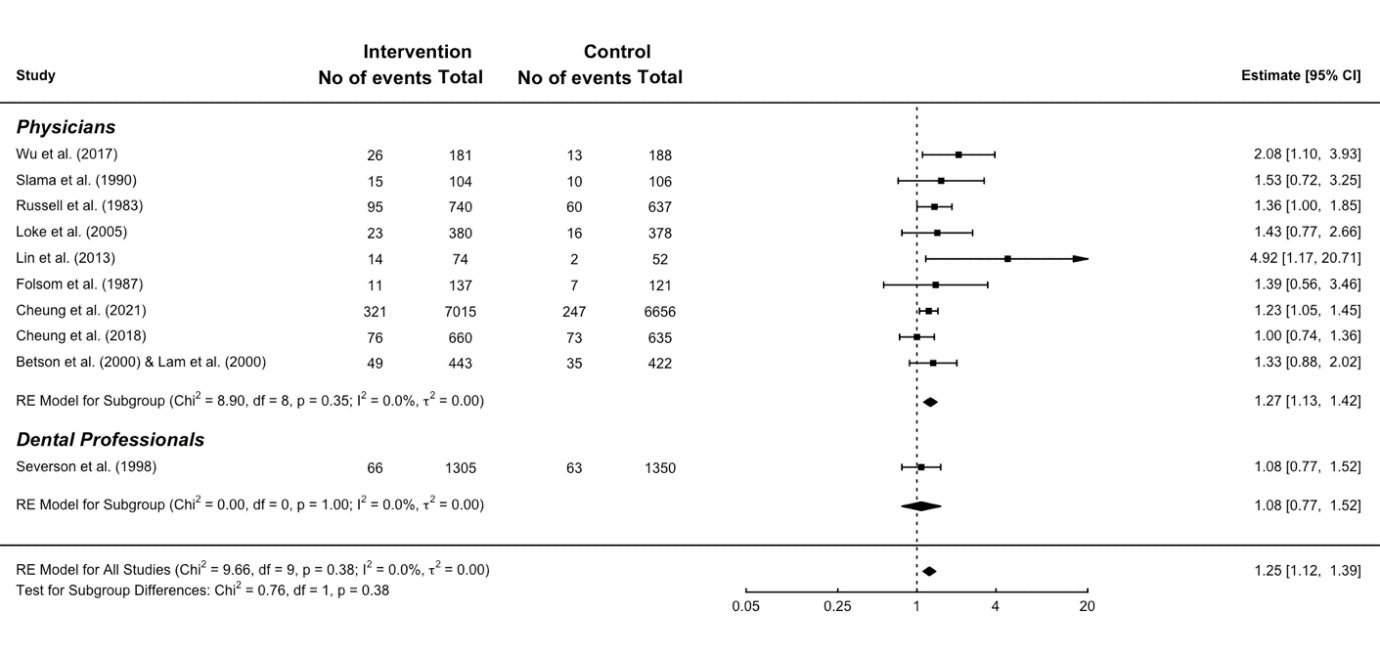


eFigure 14. Forest Plot of the average treatment effect on tobacco abstinence assessed at ≥6 months from studies of different length of advice


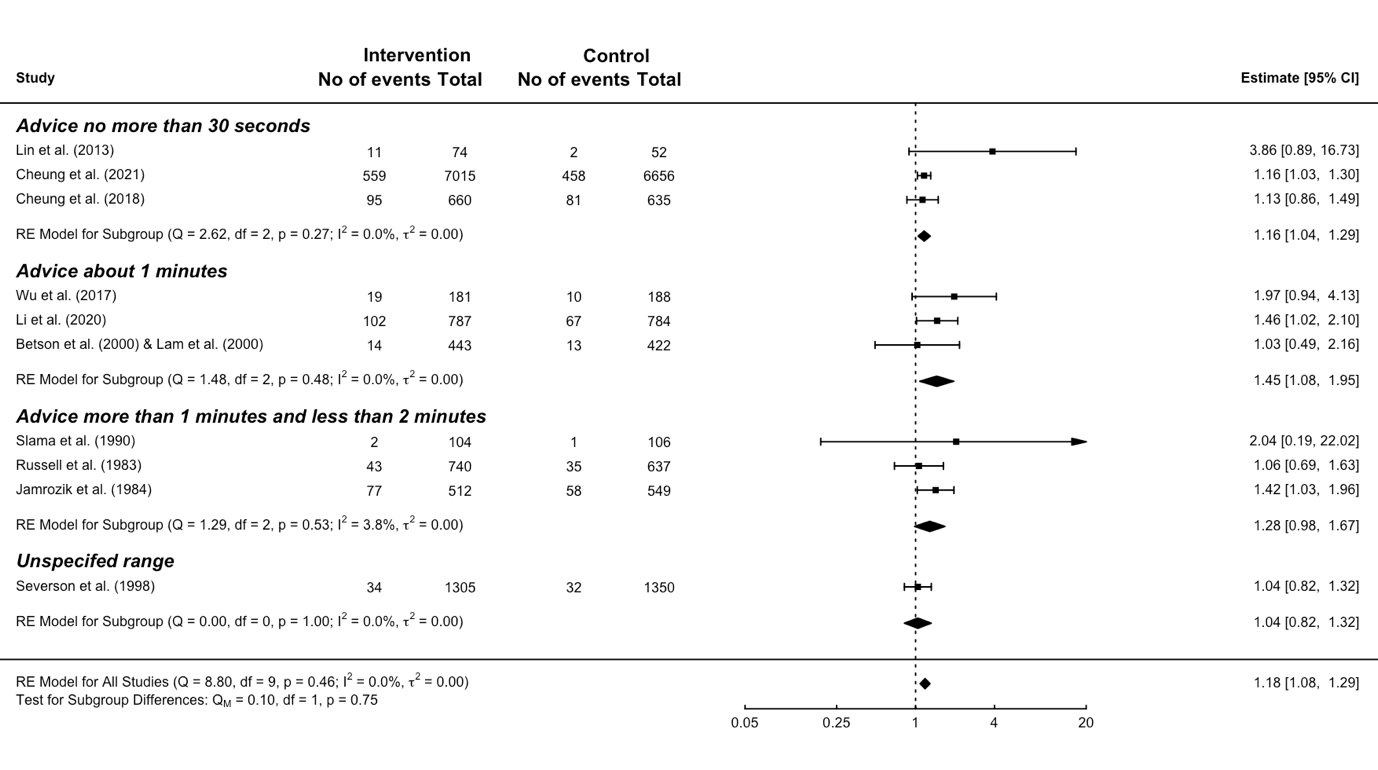


eFigure 15. Forest Plot of the average treatment effects on tobacco abstinence assessed at <6 months from studies of different length of advice
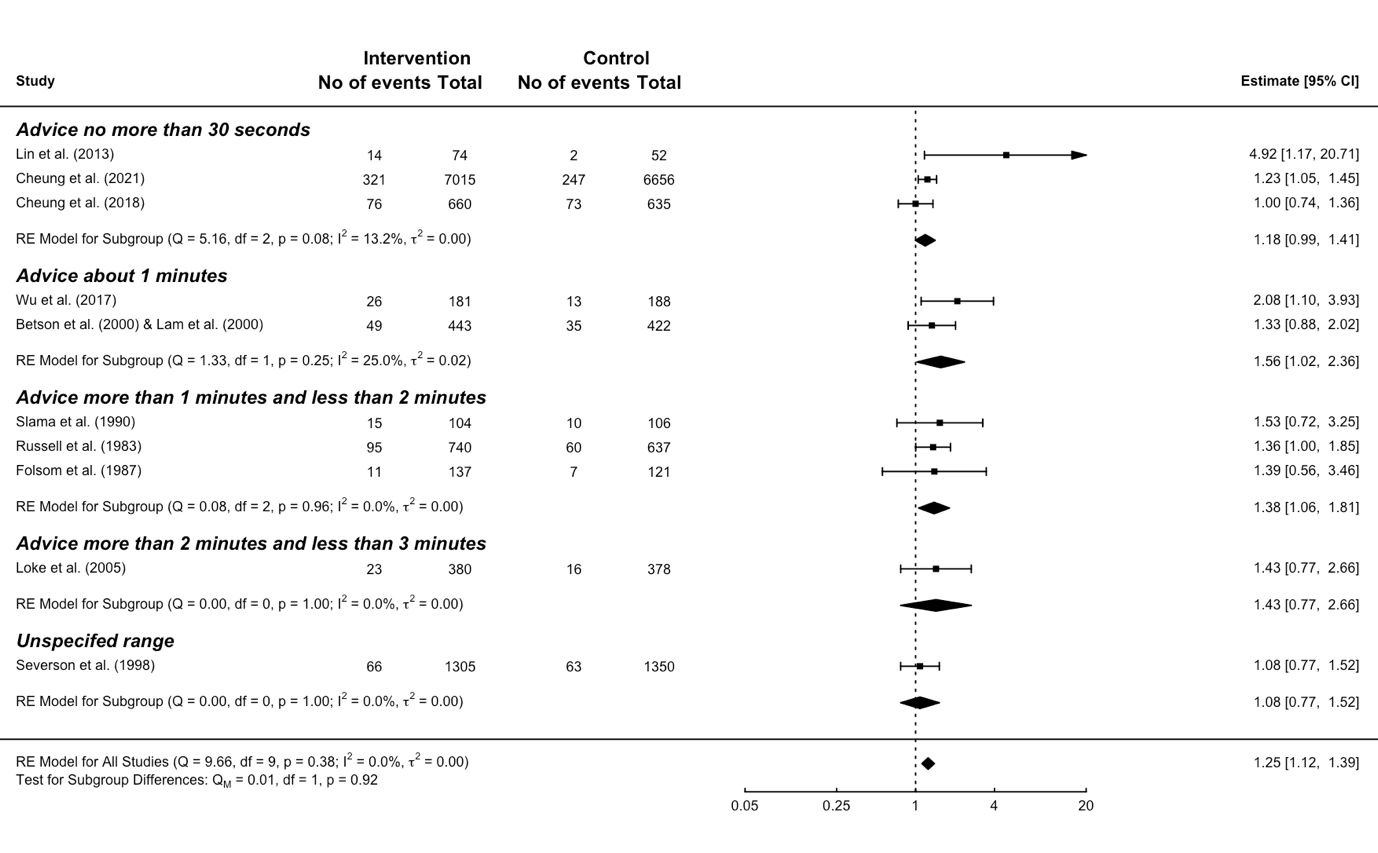


eFigure 16. Forest Plot of the average treatment effect on tobacco abstinence assessed at ≥6 months from studies of different control arms


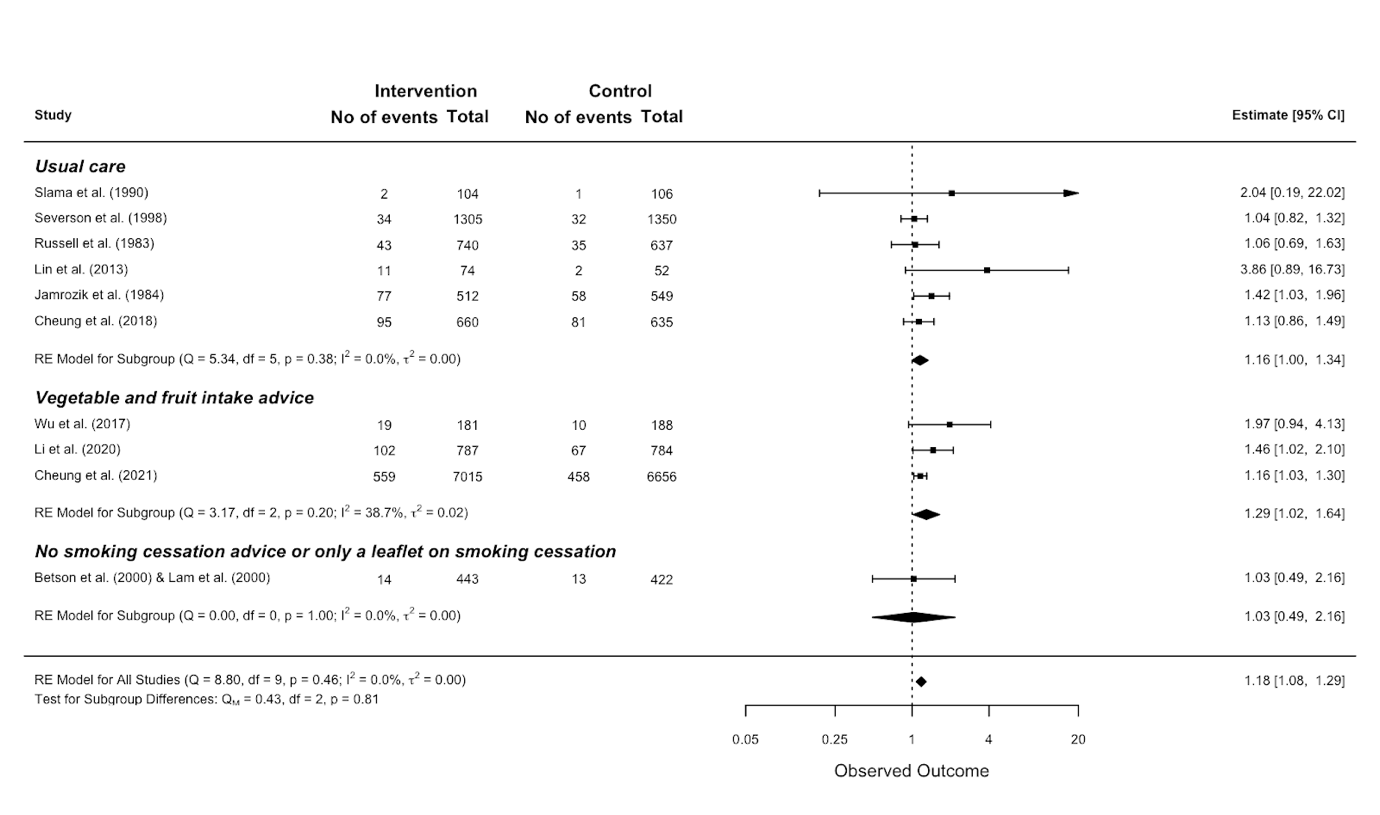


eFigure 17. Forest Plot of the average treatment effects on tobacco abstinence assessed at <6 months from studies of different control arms


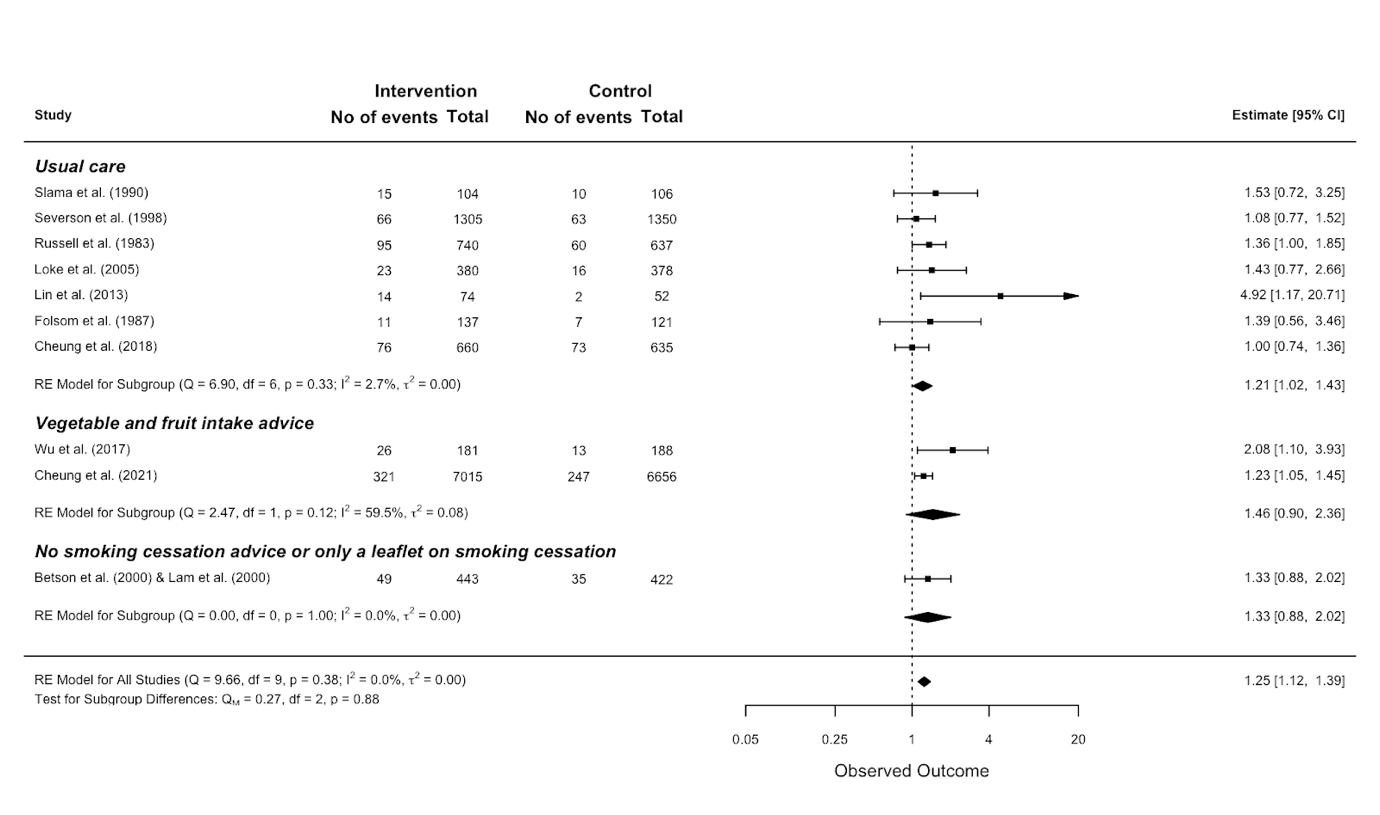


**eResults 4***. Certainty of evidence*

According to the GRADE approach, we found that the confidence interval for abstinence at ≥6 months in LMIC subgroup and quit attempts included null value, hence the certainty of evidence for abstinence at ≥6 months and quit attempts was rated down one level regarding imprecision.

Regarding indirectness of evidence, our systematic review and meta-analysis focused on self-reported abstinence and quit attempts, which were the standard outcome measures to evaluate tobacco cessation interventions. Hence, the certainty of evidence for these three outcomes were not rated down.

Risk of bias were mixed but subgroup analyses did not identify evidence of susceptible bias, and certainty of evidence for both abstinence was not rated down. Although publication bias in both abstinence outcomes were shown, adjustment for overestimation due to publication bias was done with the trim-and-fill analysis, and the certainty of evidence for both abstinence outcomes was not rated down. Regarding quit attempts, removing studies with high risk of bias changed the significant intervention effect to insignificant, hence the certainty of evidence for quit attempts outcome was rated down one level.

Moderation analyses for income level, age group and interventionists showed that the intervention effect on abstinence was not susceptible to inconsistency across subgroups. However, since we noted that different operationalization in defining quit attempts, certainty of evidence for quit attempts was rated down one level due to the inconsistency issue.

eTable 3 Summary of findings by GRADE approach

| Outcomes | Anticipated absolute effects (95% CI)* | | Relative effects (95% CI) | No. of participants (studies) | Certainty of evidence (GRADE) | Comments |
| --- | --- | --- | --- | --- | --- | --- |
|  | Control (Assumed risk) | Intervention (Corresponding risk) |  |  |  |  |
| Long-term (1-year) abstinence | 67 per 1000 | 78 per 1000 (71 to 84) | 1.17 (1.07, 1.27) | 24352 (13; included 3 imputed studies) | ⊕⊕⊕⊝^a^ | CI in Subgroup Low- and middle- income countries included null effects |
| Short-term (1 to 5 month) abstinence | 50 per 1000 | 60 per 1000 (54 to 67) | 1.20 (1.08, 1.34) | 21584 (14; included 4 imputed studies) | ⊕⊕⊕⊕^a,b^ | N/A |
| Quit attempt | 235 per 1000 | 242 per 1000 (228 to 254) | 1.03 (0.97, 1.08) | 14966 (2) | ⊕⊝⊝⊝^c,d,e^ | Only 2 good quality papers can be included. |

*The corresponding risk and its 95% CI for those receiving the intervention is calculated from the assumed risk in the untreated group and the relative effects and its 95% CI.

^a^Not downgraded due to publication bias. The overall estimate can be adjusted from trim and fill analysis (the study removed consistent with short-term abstinence).
^b^Downgraded one level due to imprecision. Confidence Intervals included insignificant effect in LMIC subgroup.

^c^Downgraded one level due to risk of bias: removing the study of high risk of bias changed the result to be insignificant.

^d^Downgraded one level due to imprecision: confidence interval included null effects.

^e^Downgraded one level due to inconsistency: measured using different operationalization.
